# Supplementary material for: A Tug-of-War between Cell Shape and Polarity Controls Division Orientation to Ensure Robust Patterning in the Mouse Blastocyst
Source: Dev Cell. 2019 Dec 2;51(5):564–574.e6. doi: 10.1016/j.devcel.2019.10.012 (PMC6892278; doi:10.1016/j.devcel.2019.10.012)
Supplement: Document S1. Figures S1–S4 and Table S1 [file mmc1.pdf]

Developmental Cell, Volume *51*

Supplemental Information

A Tug-of-War between Cell Shape and Polarity Controls Division Orientation to Ensure Robust Patterning in the Mouse Blastocyst

Ritsuya Niwayama, Prachiti Moghe, Yan-Jun Liu, Dimitri Fabrèges, Frank Buchholz, Matthieu Piel, and Takashi Hiiragi

Supplemental Figures

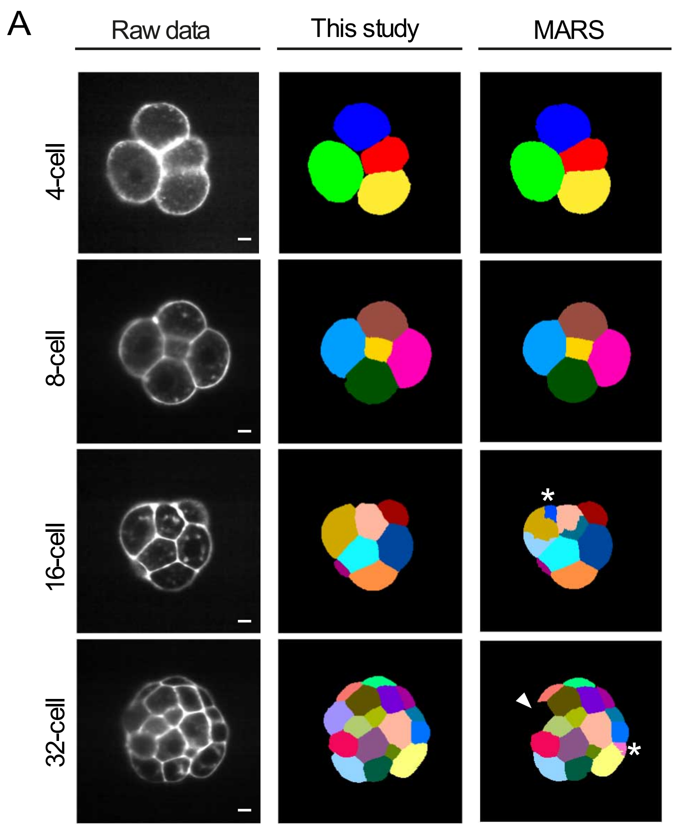

Figure S1. The fidelity of the cell membrane segmentation in this study, related to Figure 2

Representative images of the automatic membrane segmentation by the pipeline in this study and by MARS. Overall, the fidelity is comparable, with cases in which this pipeline is more optimal for segmenting the cell membrane of mouse embryos without having under-segmentation (arrowhead) or over-segmentation (stars). Scale bars: 10 μm.

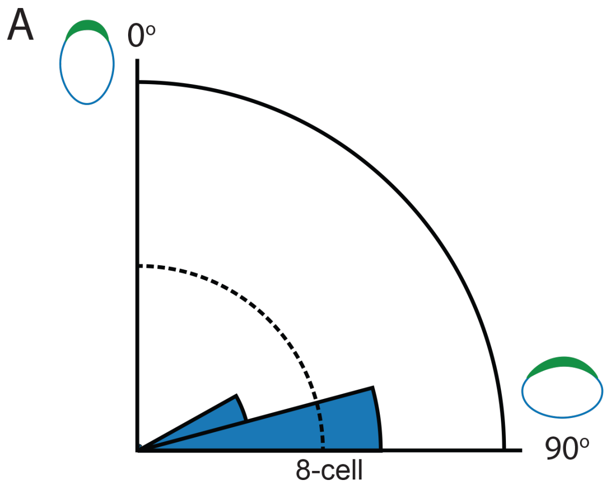

Figure S2. Relationship between cell shape and the apical domain in the 8-cell blastomeres, related to Figure 3

Distribution of the angle between the longest axis and apico-basal axis of the blastomeres in the 8-cell embryo. The solid curve represents the frequency of 1 and the dotted curve marks 0.5, with the frequency normalised to the total sample size (n=56 cells from 7 embryos), similarly to Figure 3C. While cell shape change is minimum in the 8-cell blastomeres (see Figure 3C), the apico-basal axis and the longest axis of the blastomeres are not aligned, rather they are typically perpendicular to each other. Therefore, we can clearly distinguish the impact of cell shape and the apical domain on the 8-to-16 cell divisions and conclude that it is the apical domain, but not cell shape, that controls spindle orientation during these divisions.

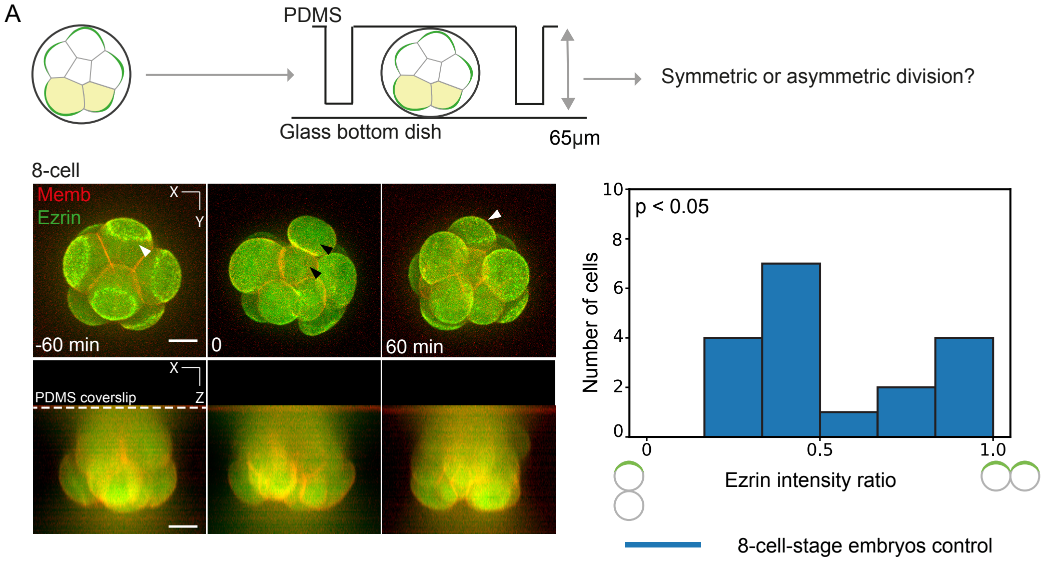

Figure S3. Cell compression of the 8-cell embryo does not change division orientation, related to Figure 4

Time-lapse images of a developing 8-cell embryo expressing Ezrin-GFP (green) and mT (red). The distribution of the Ezrin intensity ratio is significantly different from that of compressed cells in Figure 4C. p=0.039, n = 18 cells from N = 6 embryos, Kolmogorov-Smirnov test. The height of compressed embryos is 65 μm. Time 0 at the cytokinesis. White arrowheads, the apical domain. Black arrowheads, daughter cells after division. Dashed line, the compression plates. Scale bar, 10 μm.

A

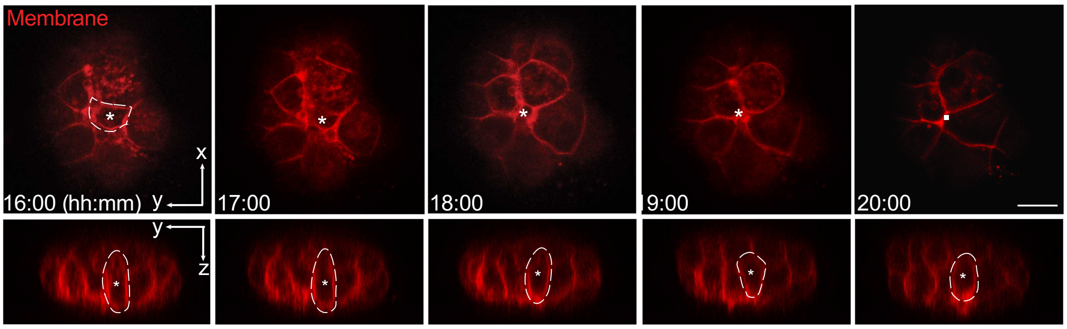

Figure S4. Cell movement as a mechanism of cell fate allocation in the blastocyst during recovery of spherical shape of the embryo, related to Figure 6

Representative images of embryos expressing mT (red) showing inward movement of cells during recovery of spherical shape of the embryo after releasing compression, contributing to inside cells. Scale bar: 20 μm.

Mouse line	Primer name	Source	Sequence	PCR product size (bp)
mTmG and mG	oIMR7318	The Jackson Laboratory, Muzumdar et al., 2007	CTCTGCTGCCTCCTGGCTTCT	Knock-in allele = 250 WT allele = 330
	oIMR7319		CGAGGCGGATCACAAGCAATA	
	oIMR7320		TCAATGGGCGGGGGTCGTT	
R26-H2B-mCherry	R26-P3	Abe et al., 2011	TCCCTCGTGATCTGCAACTCCAGTC	WT allele = 217 Knock-in allele = 270
	R26-P4		AACCCCAGATGACTACCTATCCTCC	
	R26-P6		GCTGCAGGTCGAGGGACC	
Pard6b GFP BAC	Pard6b BAC fw4	This study	TTCATATCTCGGCTCGTCCC	686
	BAC rev		AAGTCGTGCTGCTTCATGTG	
H2B-GFP	GFP-qPCR2-F	This study	CACATGAAGCAGCACGACTT	440
	GFP-qPCR3-R		CCAAGCTGAAGGTGACCAAG	

Supplemental Tables

Table S1. Genotyping primers, related to STAR Methods
