# Supplementary material for: A Tug-of-War between Cell Shape and Polarity Controls Division Orientation to Ensure Robust Patterning in the Mouse Blastocyst
Source: Dev Cell. 2019 Dec 2;51(5):564–574.e6. doi: 10.1016/j.devcel.2019.10.012 (PMC6892278; doi:10.1016/j.devcel.2019.10.012)
Supplement: Document S2. Article plus Supplemental Information [file mmc9.pdf]

Article

Developmental Cell

A Tug-of-War between Cell Shape and Polarity Controls Division Orientation to Ensure Robust Patterning in the Mouse Blastocyst

Graphical Abstract

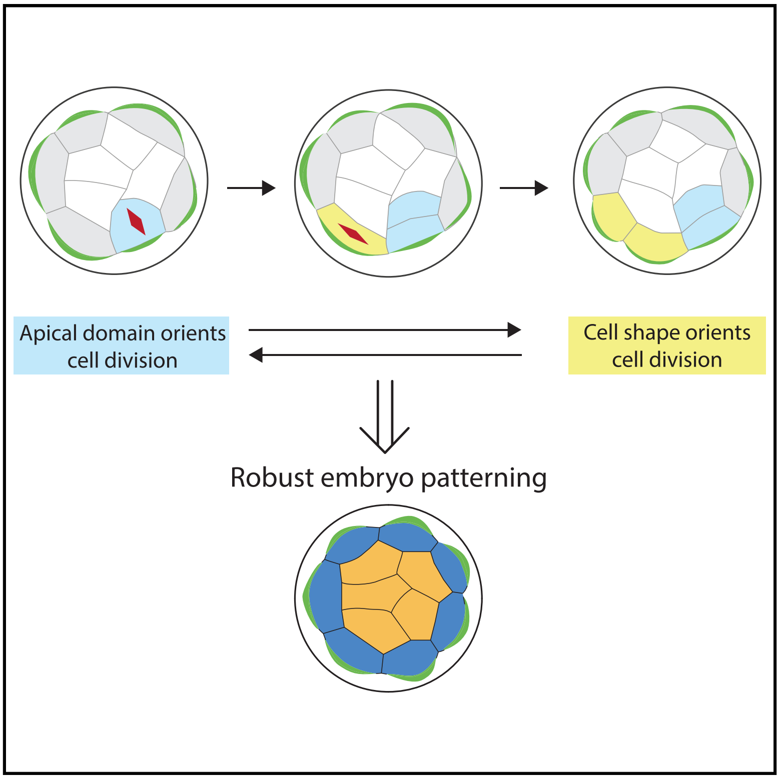

Authors

Ritsuya Niwayama, Prachiti Moghe, Yan-Jun Liu, Dimitri Fabrèges, Frank Buchholz, Matthieu Piel, Takashi Hiiragi

Correspondence

hiiragi@embl.de

In Brief

Based on systematic characterization of cell division, volume, and shape changes by *in toto* imaging, Niwayama et al. propose a model in which a tug-of-war between cell shape and the apical domain determines division orientation to ensure robust cell allocation and patterning in mouse pre-implantation embryos.

Highlights

- Cell division, volume, and shape changes are characterized by *in toto* embryo imaging
- Cell shape and the apical domain compete to determine division orientation
- Two key predictions of the model are verified experimentally
- The tug-of-war mechanism ensures robust cell allocation and patterning

Niwayama et al., 2019, Developmental Cell *51*, 564–574
December 2, 2019 © 2019 The Author(s). Published by Elsevier Inc.
https://doi.org/10.1016/j.devcel.2019.10.012

Developmental Cell
Article

A Tug-of-War between Cell Shape and Polarity Controls Division Orientation to Ensure Robust Patterning in the Mouse Blastocyst

Ritsuya Niwayama,[1] Prachiti Moghe,[1] Yan-Jun Liu,[2,5] Dimitri Fabrèges,[1] Frank Buchholz,[3] Matthieu Piel,[2] and Takashi Hiiragi[1,4,6,*]
[1]European Molecular Biology Laboratory (EMBL), Heidelberg, Germany
[2]UMR 144 Institut Curie, Institut Pierre Gilles de Gennes for Microfluidics, Paris, France
[3]Medical Systems Biology, UCC, University Hospital and Medical Faculty Carl Gustav Carus, TU Dresden, Dresden, Germany
[4]Institute for Advanced Study of Human Biology (WPI-ASHBi), Kyoto University, Kyoto, Japan
[5]Present address: Shanghai Institute of Cardiovascular Diseases and Institutes of Biomedical Sciences, Zhongshan Hospital, Fudan University, Shanghai, China
[6]Lead Contact
*Correspondence: hiiragi@embl.de
https://doi.org/10.1016/j.devcel.2019.10.012

SUMMARY

Oriented cell division patterns tissues by modulating cell position and fate. While cell geometry, junctions, cortical tension, and polarity are known to control division orientation, relatively little is known about how these are coordinated to ensure robust patterning. Here, we systematically characterize cell division, volume, and shape changes during mouse pre-implantation development by *in toto* live imaging. The analysis leads us to a model in which the apical domain competes with cell shape to determine division orientation. Two key predictions of the model are verified experimentally: when outside cells of the 16-cell embryo are released from cell shape asymmetry, the axis of division is guided by the apical domain. Conversely, orientation cues from the apical domain can be overcome by applied shape asymmetry in the 8-cell embryo. We propose that such interplay between cell shape and polarity in controlling division orientation ensures robust patterning of the blastocyst and possibly other tissues.

INTRODUCTION

Tissue patterning is driven by position-dependent differentiation, coordinated movement, and division of cells. In mammalian embryos, the first cell lineage segregation results in the formation of the blastocyst in which an inner cell mass (ICM) is surrounded by outer trophectoderm (TE) cells (Rossant and Tam, 2009; Yamanaka et al., 2006). This position-dependent cell fate specification has been studied for decades (Tarkowski and Wróblewska, 1967). Our recent study showed that asymmetry in cell contacts directs the formation of an apical domain on the contact-free surface of outside cells and that this polarized domain is functionally required and sufficient for TE differentiation (Korotkevich et al., 2017). Cell position in turn, can be controlled by cell division and movement. For instance, inside cells in the early mouse embryo are generated by asymmetric divisions (Anani et al., 2014; Johnson and Ziomek, 1981) as well as inward cell sorting (Watanabe et al., 2014) driven by differential cortical tension (Maître et al., 2016; Samarage et al., 2015). While the orientation of cell division per se does not determine cell fate (Korotkevich et al., 2017), it influences how the less-contractile apical domain is segregated between daughters, hence their cell sorting behavior and positioning within the embryo (Maître et al., 2016).

While *in toto* live-imaging and lineage tracking established that the lineage tree and division patterns of the early mouse embryo is non-stereotypic (Kurotaki et al., 2007; Morris et al., 2010; Strnad et al., 2016), the number of inside (and ICM) and outside (and TE) cells in an embryo at a given time is controlled with relatively little variability (Dietrich and Hiiragi, 2007; Saiz et al., 2016; Watanabe et al., 2014). Therefore, a key open question for blastocyst patterning is how these numbers are controlled within each embryo and, specifically, whether spatially coordinated cell divisions contribute to this robust patterning.

The orientation of cell division is influenced by cell geometry. In many cell types, the division plane bisects the longest axis, according to Hertwig's rule (Dumollard et al., 2017; Hertwig and Hertwig, 1884). Microtubules are proposed to sense cell shape by exerting pulling forces that scale to microtubule length (Minc et al., 2011; Pierre et al., 2016). Epithelial tricellular junctions may also act as cell shape sensors (Bosveld et al., 2016). It has recently been shown, however, that cortical tension can override cell geometrical cues in some tissues to control division orientation (Campinho et al., 2013; Finegan et al., 2019; Scarpa et al., 2018; Wang et al., 2017). Likewise, cell polarity is also known to control the orientation of cell division. In intestinal epithelial (Caco-2) cells, cortical Ezrin positions the centrosome and thereby controls division orientation (Hebert et al., 2012). Similarly, in the 8-cell stage mouse embryo, the apical domain drives its asymmetric segregation between daughter cells by tethering one of the spindle poles, or microtubule organizing centers, to the sub-apical region (Korotkevich et al., 2017).

 © 2019 The Author(s). Published by Elsevier Inc.
This is an open access article under the CC BY license (http://creativecommons.org/licenses/by/4.0/).

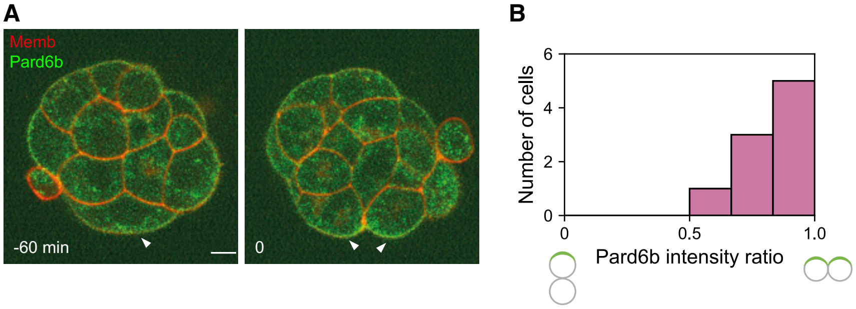

Figure 1. The 16- to 32-Cell Divisions Are Predominantly Symmetric

(A) Live-imaging of mouse embryos expressing mT (red); Pard6b-GFP (green) undergoing the 16–32 cell division. Note that the apical domain (arrowheads) is segregated symmetrically upon division. Time 0 corresponds to the cytokinesis. Scale bar, 10 μm. See also Video S1.

(B) Segregation of the apical domain upon 16–32 cell division, as measured by the inheritance of the Pard6b signal intensity. The majority of outside cells segregate the apical domain symmetrically between daughters. n = 9 cells.

Nevertheless, relatively little is known about how these mechanisms are coordinated in developing tissues to achieve robust morphogenesis and patterning.

In this study, we use early mouse embryos to investigate how cell division patterns are regulated by different mechanisms to ensure proper cell fate allocation and tissue patterning.

RESULTS

The Orientation of Cell Divisions Markedly Differs between the 8–16 and 16–32 Cell Divisions in the Mouse Embryo

Recent studies from us and others showed that the majority of 8–16 cell divisions result in the asymmetric segregation of the apical domain between daughter cells (Anani et al., 2014; Korotkevich et al., 2017; Watanabe et al., 2014). This, followed by cell sorting, results in 16-cell embryos with 0 to 4 inside cells, as defined in the present study and others (Anani et al., 2014; Dietrich and Hiiragi, 2007; Graham and Lehtonen, 1979; Watanabe et al., 2014) as those lacking any embryonic outer surface. As additional inner cells are generated by subsequent divisions, we investigated whether similar mechanisms may be at work during 16–32 cell divisions. The live-imaging analysis showed that in contrast to the preceding stage, most cells undergo symmetric divisions during the 16–32 cell transition (Figures 1A and 1B; Video S1), in agreement with an earlier study (Watanabe et al., 2014). Since this occurs despite the persistence of the apical domain, we investigated the mechanism underlying this abrupt change in cell division pattern.

A Pipeline to Systematically Characterize Cell Shape, Division Pattern, and Lineage by Digital Reconstruction of the Embryonic Development

To study cell division control and its relationship with cell fate specification, we developed a pipeline to systematically characterize cell shape, division pattern, and lineage in developing mouse embryos (Figure 2A). Pre-implantation development of transgenic mouse embryos expressing H2B-mCherry;mTmG is monitored by *in toto* imaging using an inverted light-sheet microscope (InVi-SPIM, Strnad et al., 2016), scanning with 1 μm z-interval every 10 min. From resultant images, the nuclear signal was used to track lineage as well as to initiate membrane segmentation using Ilastik and Level-set (Mikula et al., 2011). This pipeline allowed semi-automatic digital reconstruction of embryonic development for 7 mouse embryos from the 4/8-cell to the 32-cell stage. Its performance was comparable to that of an earlier image-processing method, multiangle image acquisition, 3D reconstruction and cell segmentation (MARS) (Fernandez et al., 2010), and optimized for mouse embryo images (Figure S1; Video S2).

The digital reconstruction provided comprehensive information on cell shape, division pattern, and lineage, with cell fate judged by its eventual position, either inside (ICM) or outside (TE) of the blastocyst. Cell lineage analysis confirmed that the lineage segregation pattern is not stereotypic in early mouse embryos (Figure 2B) (Strnad et al., 2016; Watanabe et al., 2014). While individual cell volume is significantly larger for outside cells than inside cells (Figure 2C, n = 37 and 74 for inside cells and outside cells, respectively, $p = 2.5 \times 10^{-15}$), the total embryo volume remains constant until the 32-cell stage (Figure 2D, p = 0.059), indicating that the embryo undergoes cleavages without tissue growth during pre-implantation development, in agreement with an earlier study (Aiken et al., 2004).

Cell Shape Changes in Outside Cells of the 16-Cell Embryo that Undergo Symmetric Divisions

Cell shape changes substantially in outside cell of the 16-cell embryo, reflecting the higher cortical tension on the embryo surface after compaction (Figure 3A) (Maître et al., 2015). Those 16-cell-stage outside cells with higher aspect ratio (2.1 on average, n = 74) undergo cytokinesis bisecting the longest axis, in contrast to other cells where division planes are random (Figures 3B and 3C, $p = 0.56, 0.38, 2.0 \times 10^{-9}$ for the 8-cell-stage, 16-cell-stage inside and 16-cell-stage outside cells, respectively). These data confirm our earlier finding that the majority of outside cells at the 16-cell stage undergoes symmetric divisions, as judged by the segregation of the apical domain (see Figure 1B).

A Tug-of-War between Cell Shape and Polarity Controls Division Orientation

Taken together, these data show that when cell shape asymmetry is weak (as in the 8-cell embryo), the apical domain directs division orientation, whereas cells with geometrical asymmetry (outside cells in the 16-cell embryo) bisect their longest axis in accordance with Hertwig's rule despite the presence of the apical domain (Hertwig and Hertwig, 1884). Clearly, there are stages of embryonic development during which division orientation cannot be explained solely by either the apical domain (the 16-cell stage, see Figure 1) or cell shape (the 8-cell stage, Figure S2). These findings suggest that a tug-of-war between cell shape and polarity controls division orientation, and the stronger

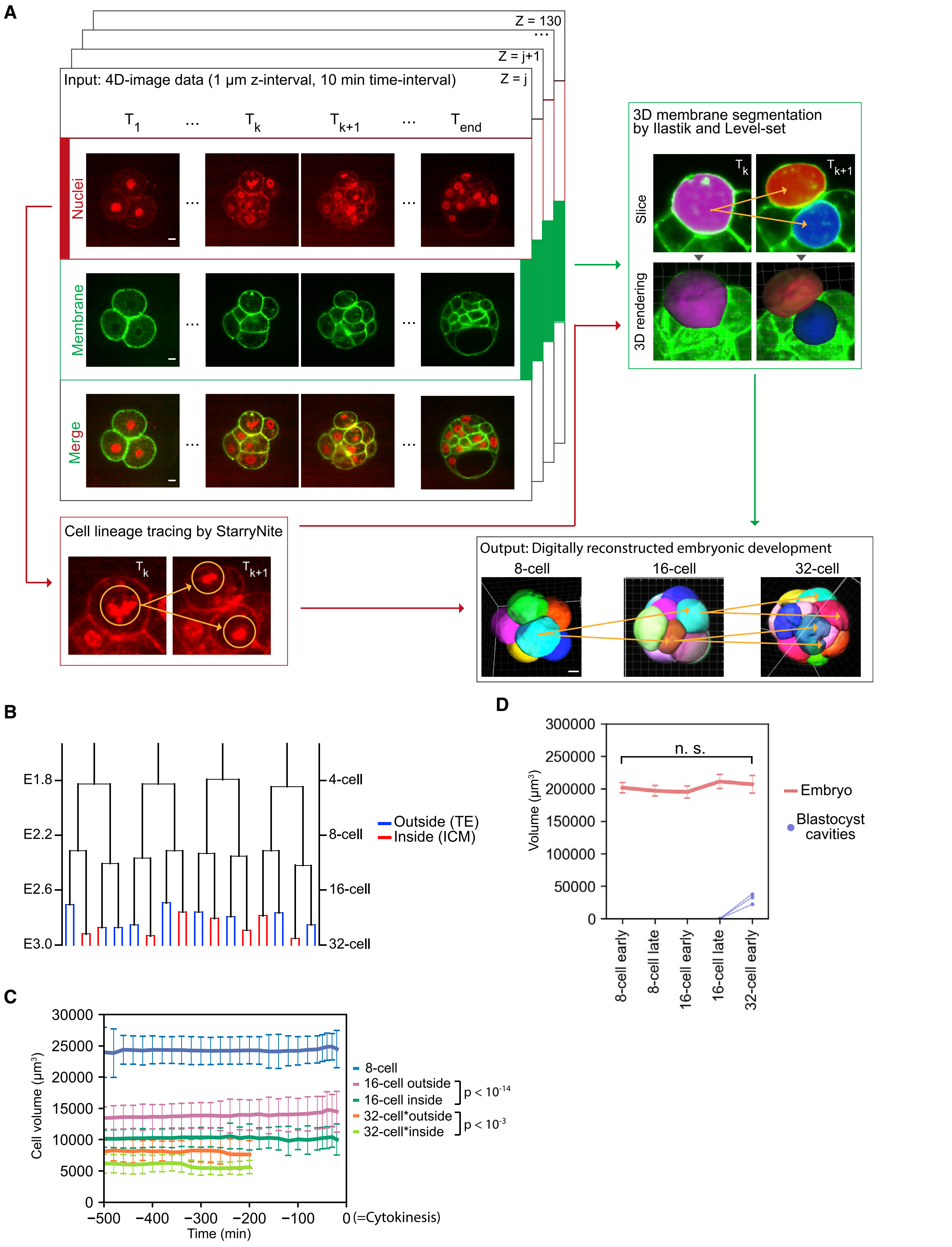

(legend on next page)

cue dominates the control when the directed orientations are in conflict.

This model makes two experimentally testable predictions on division orientation in the mouse pre-implantation embryo. First, when outside cells of the 16-cell-stage embryo are released from geometrical asymmetry, their division orientations would be controlled by the apical domain. Namely, they would change their division pattern from predominantly symmetric to asymmetric. Second, when cell shape asymmetry is introduced to cells of the 8-cell embryo, division orientation will follow Hertwig's rule despite the presence of the apical domain, possibly inducing symmetric rather than asymmetric 8–16 cell divisions.

Release of Cell Shape Asymmetry Shifts Division Orientation toward Cell-Polarity-Guided Asymmetric Division

We experimentally tested the first prediction by isolating blastomeres from 16-cell embryos and thus relieving their shape asymmetry. Upon 16–32 cell-stage division, the majority of the cells segregated the apical domain asymmetrically between daughter cells (Figure 4A; Video S3, n = 27, the distribution is significantly different from that in Figure 1B, $p = 2.0 \times 10^{-4}$), in agreement with the prediction.

Introduction of Cell Shape Asymmetry Shifts Division Orientation toward Cell-Shape-Guided Symmetric Division

Next, we tested the second prediction of the model by changing the geometry of 8-cell blastomeres. First, we isolated blastomeres from 8-cell embryos and compressed individual cells with a microfluidics device. We controlled the compression such that the aspect ratio of cells were 2.8 on average, a value comparable to that of outside cells of the 16-cell embryo. The plane of division in these compressed cells bisected the longest axis, and we observed symmetric segregation of the apical domain positioned at the stretched surface (Figure 4B; Video S4; n = 8, 10 for compressed and control cells, respectively, p = 0.0038).

Furthermore, we compressed the whole embryo to change cell geometry, in particular, those facing the stretched embryo surface. When 8-cell embryos were compressed to a height of 20 μm, the aspect ratio of individual cells reached 3.0 on average (N = 15 embryos, Figure 4C; Video S5). Under this condition, these cells underwent symmetric divisions, as judged from symmetric segregation of the apical domain (n = 18 cells, $p = 6.2 \times 10^{-5}$, in comparison to control cells in Figure 4B). Mild compression of the 8-cell embryo did not change division orientation, indicating that cell shape, but not the compression itself, directs the orientation of cell divisions (Figure S3).

Together, these data are in agreement with the predictions and experimentally support the model in which the stronger cue between cell shape and polarity controls division orientation when the directed orientations are in conflict.

Cell Division Pattern Does Not Determine Cell Fate in the Blastocyst

The compression of the 8-cell embryo changed the pattern of 8–16 cell divisions from predominantly asymmetric to symmetric (Figure 4C). In the normal embryo, the majority (86%) of the blastomeres differentially segregate the apical domain between the daughter cells with the resulting Ezrin intensity ratio lower than 0.33 (Korotkevich et al., 2017), whereas this is the case only for one of 18 cells (5.6%) in compressed embryos (N = 15 embryos). This presented us with an interesting opportunity to examine whether cell division pattern per se determines cell fate specification in the mouse blastocyst. We monitored development of the embryo after releasing compression and examined cell fate specification in the resulting blastocyst (Figure 5A). Notably, these embryos developed into blastocysts with total cell number and ICM/TE proportions that were comparable to control embryos, with no significant difference in their spatial distribution (Figures 5B and 5C). This clearly demonstrates that cell division pattern per se does not determine cell fate in the mouse blastocyst, in agreement with findings in our earlier study (Korotkevich et al., 2017).

The Tug-of-War Mechanism Ensures Robust Cell Allocation and Patterning in the Mouse Blastocyst

The finding that embryos form normal blastocysts despite temporary geometric deformation prompted us to investigate the mechanisms underlying the robust control of cell position allocation and fate specification. No discernible inside cells were observed in embryos compressed during the 8-cell to 16-cell stage, due to geometrical constraints (Figure 6A). Upon release from compression, the height of the embryos progressively increased over 16 h from 48 to 82 μm to recover their spherical shape (Figures 6B and 6C; Video S6). Notably, the number of inner cells in compressed embryos became comparable to control embryos after 16 h of recovery (Figure 6D). The number of inside cells increased from 11 to 22 cells on average in the control embryos developing for 17 h, while the total cell number increased

Figure 2. A Pipeline for Digital Reconstruction of the Mouse Pre-implantation Development to Systematically Analyze Cell Geometry, Division, and Lineage

(A) An image-processing pipeline digitally reconstructs mouse pre-implantation development, using a nuclear signal to track lineage and initiate membrane segmentation. Scale bars, 10 μm. See also Video S2.

(B) A representative cell lineage tree from the 4-cell to 32-cell stage built from the analysis. Blue and red line indicate cells that are positioned at the outside (TE) or inside (ICM) the embryo, respectively, by the 32-cell stage.

(C) Change in cell volume. Time 0 cytokinesis. Cell position in the 16-cell and 32-cell embryos are classified according to its position before the entry into mitosis. n = 56, 37, and 74 cells in 7 embryos for the 8-cell-stage embryo, 16-cell-stage inside and 16-cell-stage outside cells, respectively; and n = 12 and 28 cells in 4 embryos for the 32-cell-stage inside and outside cells, respectively. The volume of inside cells is significantly smaller than the outside cells at the 16-cell stage and 32-cell stage with $p = 2.5 \times 10^{-15}$ and 3.7×10^{-4}, respectively, by Student's t test. Note that for the 32-cell stage, data are not available until the cytokinesis, therefore, time is shown with -500 as the start of the 32-cell stage, differently from the other stages. Error bars, the standard deviation.

(D) The total embryo volume does not change from the 8-cell to 32-cell stage. p = 0.059, one-way ANOVA-test. n = 6 embryos. Blue lines indicate the total volume of the blastocyst cavities for those embryos (n = 3) that initiated cavitation. Error bars, the standard deviation.

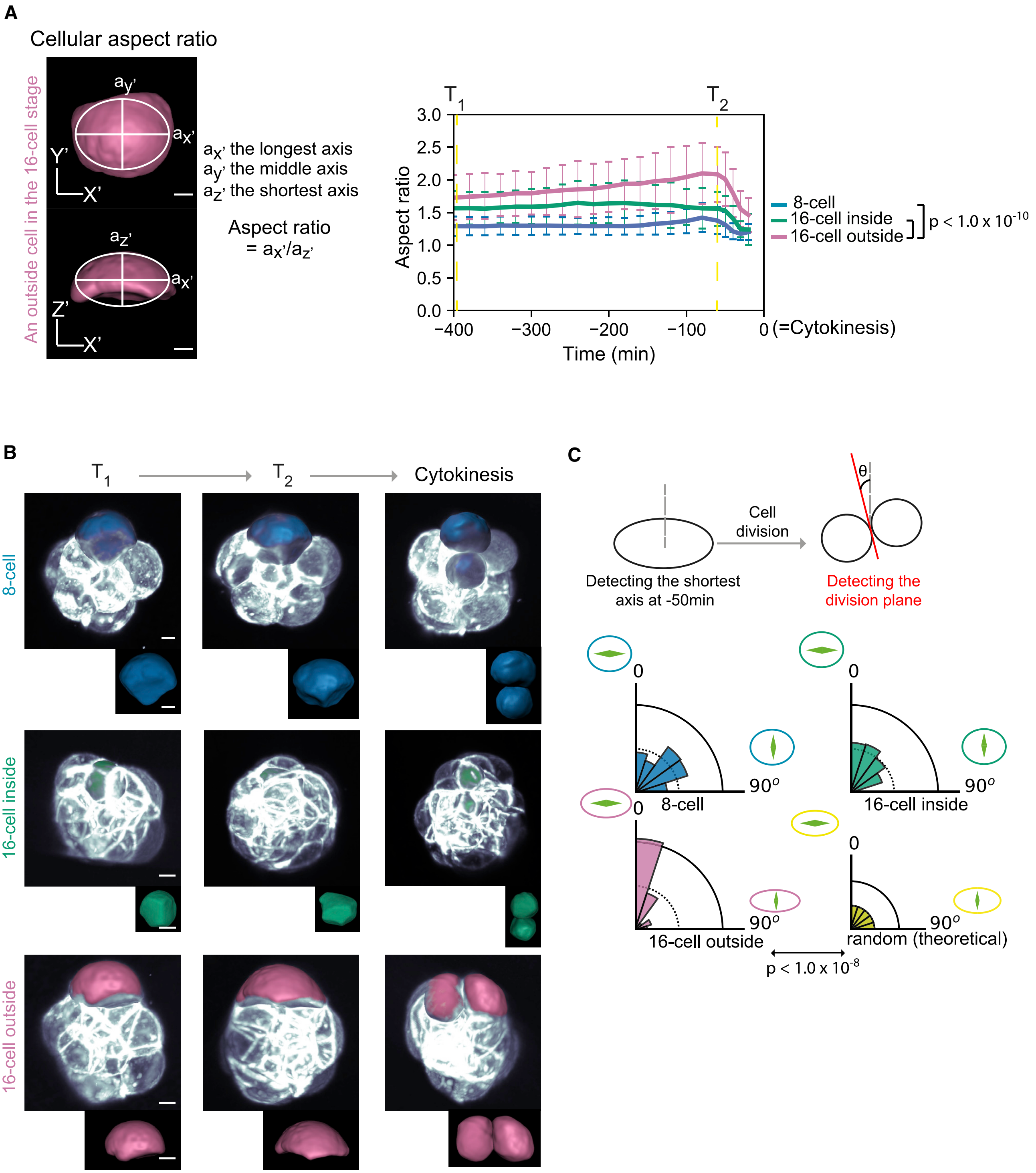

Figure 3. Cell Geometry Changes in Outside Cells of the 16-Cell Embryo that Undergo Symmetric Divisions

(A) Measurement and tracking of cellular aspect ratio. At each time-point, the longest, middle, and shortest axes of cells are measured, and the ratio of the longest to shortest is calculated. Time 0 corresponds to the cytokinesis. The aspect ratio is significantly higher in the 16-cell-stage outside cells than the other cells, $p < 1.0 \times 10^{-10}$. Dunnett's test. n = 56, 37, 74 in 7 embryos for the 8-cell, 16-cell inside and 16-cell outside cells, respectively. Scale bars, 10 μm. Error bars, the standard deviation.

(B) A representative cell geometry at T1, T2 (400 and 60 min prior to the cytokinesis, respectively) and at the cytokinesis. Scale bars, 10 μm.

(C) Distribution of the division planes. The division planes of the 16-cell outside cells are preferentially aligned to the shortest axis of the cell, significantly different from random distribution, $p < 1.0 \times 10^{-8}$. Kolmogorov-Smirnov test. n = 56, 37, and 73 in 7 embryos for the 8-cell-stage, 16-cell-stage inside and 16-cell-stage outside cells, respectively.

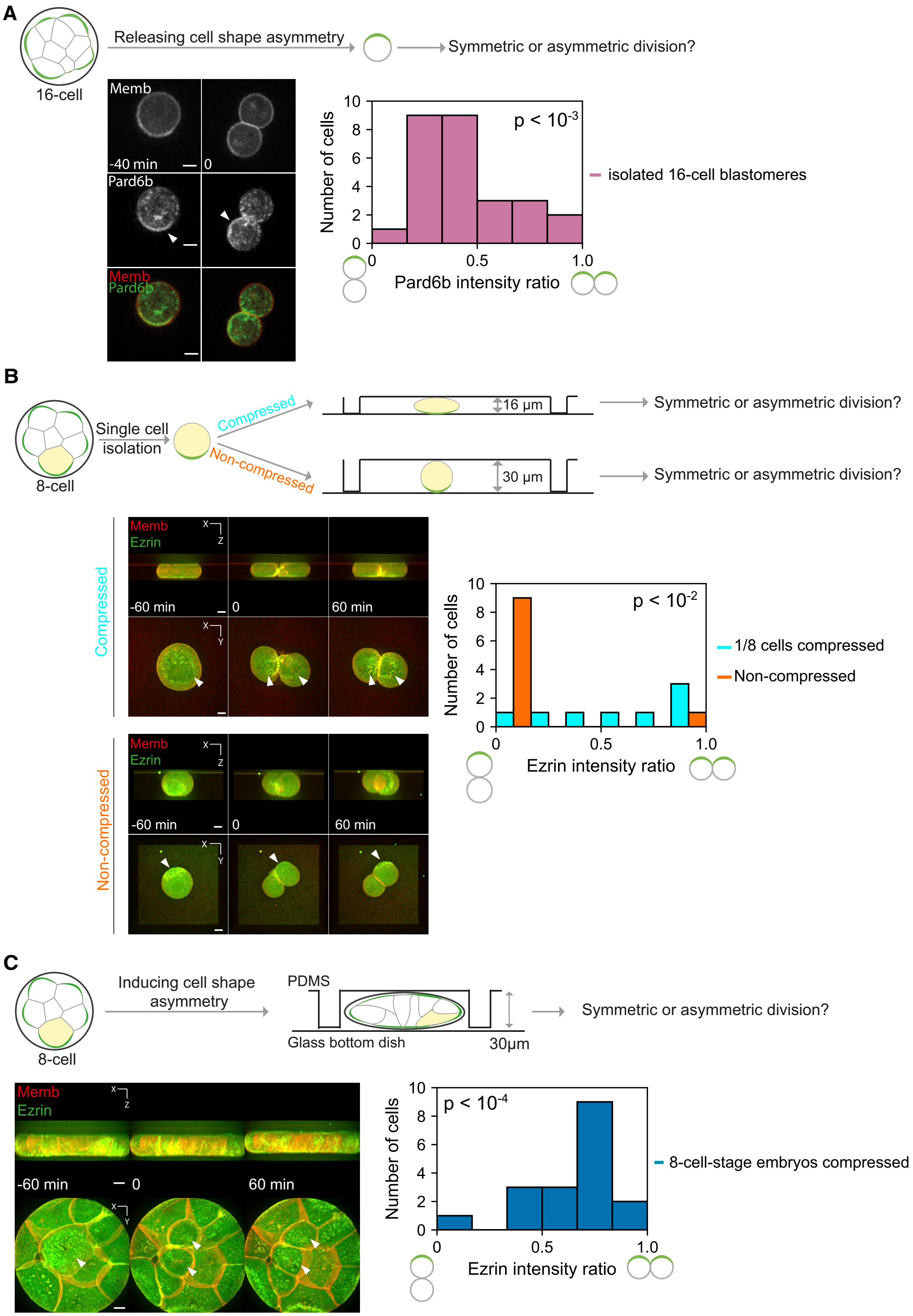

Figure 4. Manipulating Cell Geometry Changes Its Influence on Division Orientation

(A) The release of cell shape asymmetry in the 16-cell-stage blastomeres changes their division orientation. Time-lapse images of a developing 16-cell-stage blastomere expressing Pard6b-GFP (green) and mT (red), with Time 0 at the cytokinesis, see also Video S3. The distribution of the Pard6b intensity ratio between daughters is significantly different from that of the whole embryos (Figure 1B). $p = 2.0 \times 10^{-4}$, n = 27, Kolmogorov-Smirnov test. Scale bars, 10 μm.

(legend continued on next page)

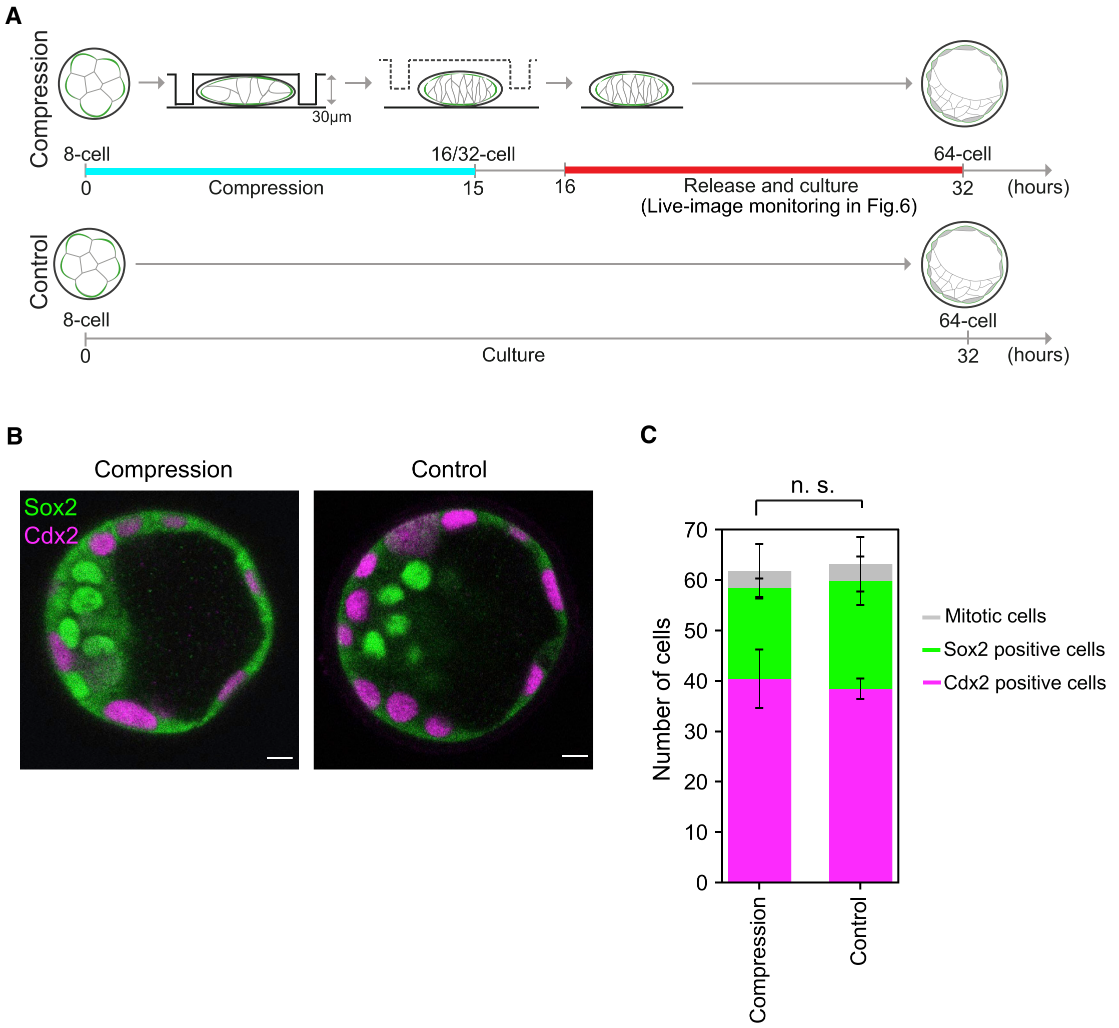

Figure 5. Cell Division Pattern Does Not Influence Cell Fate in the Blastocyst

(A) The experimental design to examine whether changes in the 8–16 cell division pattern influences cell fate in the blastocyst and to monitor the generation of inner cells in the blastocyst during recovery (Figure 6). Time 0 corresponds to the start of compression.

(B) Immunofluorescence staining for Sox2 (green) and Cdx2 (magenta) of the blastocysts cultured with (Compression) or without (Control) compression between the 8 and 16 cell stages. Scale bars, 10 μm.

(C) The number of Cdx2 (magenta) and Sox2 (green) positive cells in the blastocysts cultured with (Compression) or without (Control) compression. The number of mitotic cells is shown in gray. The total number of cells, as well as the number of Cdx2 and Sox2 positive cells remain unchanged, with $p = 0.69$, 0.38, and 0.14, respectively, using Student's t test. $n = 9$, 7 for compressed and control embryos. Error bars, the standard deviation.

from 32 to 62 cells (N = 4 embryos). In contrast, when embryos were recovering from compression, the number of inside cells rapidly increased from 5 cells, remarkably reaching a number (25 cells on average, N = 3) comparable to that of the normal embryos in the mature blastocyst. The total number of cells in the blastocyst were also comparable to control embryos (65 cells on average, N = 3 embryos).

To understand the mechanism of the rapid increase in the inside cells after the release of compression (see Figure 6D), we specifically examined the first divisions in control and

(B) The introduction of cell shape asymmetry in the 8-cell blastomeres changes their division orientation. Cells are compressed in a microfluidics device with its pillar height 16 and 30 μm for experimental and control (non-compressed) groups, respectively. The average height and diameter of compressed cells are 17 and 46 μm, respectively, with the aspect ratio 2.8. Time-lapse images of a developing 8-cell-stage blastomeres expressing Ezrin-GFP (green) and mT (red), see also Video S4. Distribution of the Ezrin intensity ratio is significantly different between compressed and non-compressed (control) cells. $p = 3.8 \times 10^{-3}$, $n = 8$, 10 for compressed and control cells, respectively, Kolmogorov-Smirnov test. Scale bars, 10 μm.

(C) Cell shape asymmetry induced by compression of the 8-cell embryos changes division orientation. The height and the diameter of compressed embryos are 20 and 110 μm, respectively. Time-lapse images of a developing 8-cell embryo expressing Ezrin-GFP (green) and mT (red) see also Video S5. The distribution of the Ezrin intensity ratio is significantly different from that of non-compressed cells in Figure S3. $p = 6.2 \times 10^{-5}$, $n = 18$ cells from N = 15 embryos, Kolmogorov-Smirnov test. Arrowheads, the apical domain. Scale bars, 10 μm.

CellPress

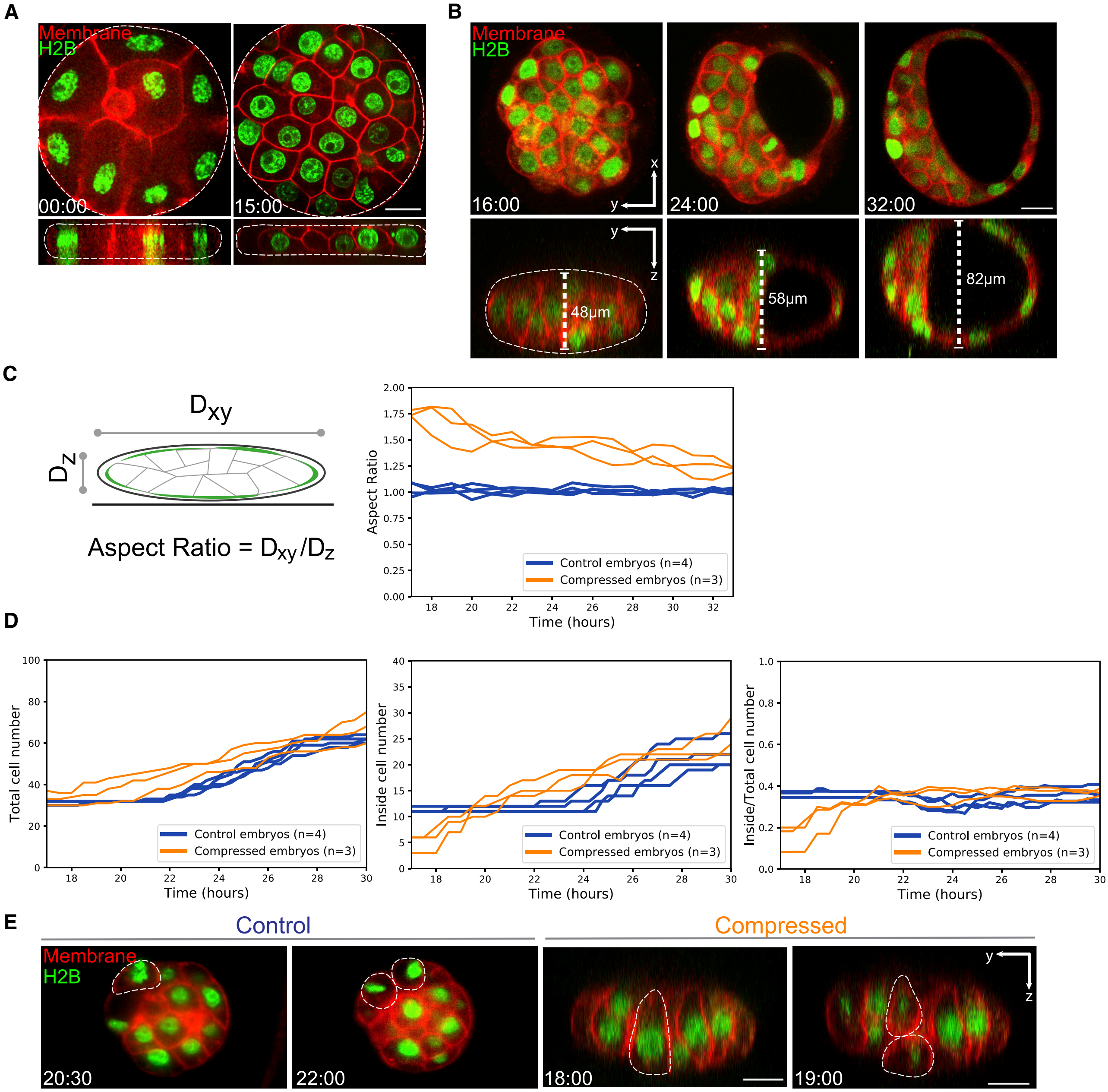

Figure 6. Analysis of Cell Fate Allocation in the Blastocyst during Recovery of Spherical Shape of the Embryo

(A) Representative images of compressed embryos expressing H2B-GFP (green) and mT (red) highlighting embryo shape and cell numbers at t = 00:00 (start of compression at the compacted 8-cell stage, hh:mm) and t = 15:00 (end of 15-h compression). Top panel shows xy-plane and bottom panel shows orthogonal yz-plane. Scale bars, 20 μm.

(B) Time-lapse images from XY- (top) and YZ-plane (orthogonal) of developing embryos after releasing compression, see also Video S6.

(C) Change in the aspect ratio of the embryos under different experimental conditions. n = 4, 3 for non-compressed (control), and compressed embryos, respectively.

(D) Change in the total cell number, inner cell number, and ratio of inner to total cell number in the embryos after releasing compression. n = 4, 3 for non-compressed (control), and compressed embryos, respectively.

(E) Representative images showing the orientation of cell division during the 32–64 cell transition stage after releasing compression to increase number of inner cells, highlighting cell geometry prior to cell division; see also Video S7. Scale bars, 20 μm.

compressed embryos following the release. We found that many cells in the compressed-and-released embryos underwent asymmetric divisions generating inside cells, whereas many of the outside cells in control of 32-cell stage embryo underwent symmetric divisions (Figure 6E; Video S7). In addition, cells in the compressed-and-released embryos moved toward the interior of the embryo during the recovery of the spherical shape (Figure S4). Notably, cells in the

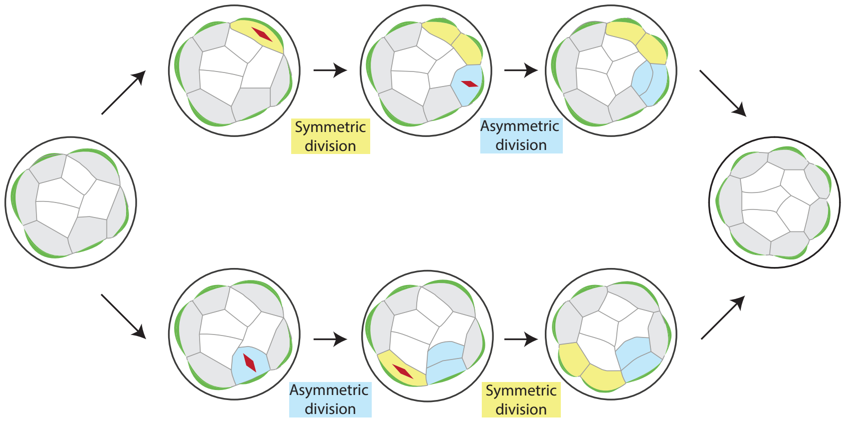

Figure 7. Schematic Representation of the Mechanism for Proper Tissue Compartmentalization by Controlling Division Orientation
Cells stretched along the embryo surface undergo symmetric division, inducing other cells on the surface to change shape accordingly and divide asymmetrically (top). Conversely, cells with a longer apico-basal axis undergo asymmetric division guided by the apical domain, thereby stretching neighboring cells and prompting them to divide symmetrically (bottom). Eventually, this interplay between cell shape and polarity ensure appropriate partitioning of ICM and TE cells and robust patterning of the blastocyst.

compressed-and-released embryos had an apico-basally elongated shape that would induce asymmetric division, while outside cells in the normal 32-cell stage embryo had a perpendicular geometrical asymmetry, similar to the 16-cell stage outside cells (see Figures 3B and 3C), that drives symmetric division. These findings are in line with the tug-of-war model and suggest that this mechanism of controlling division orientation provides a fail-safe mechanism to ensure that certain proportions of cells are allocated to ICM or TE in the mouse blastocyst.

DISCUSSION

In this study, we comprehensively characterized cell division pattern as well as cell volume and shape changes during mouse pre-implantation development using a newly developed image-processing pipeline. The analysis led us to propose a model in which a tug-of-war between cell polarity and shape determines the orientation of cell division. When the orientation directed by the two cues are in conflict, the stronger cue dominates. Two key predictions of the model were verified experimentally: when outside cells of the 16-cell embryo are released from shape asymmetry, they undergo asymmetric divisions controlled by the apical domain, in contrast to the normal embryo in which the majority of outside cells divide symmetrically to generate two outside cells. When cells of the 8-cell embryo are compressed, cell shape asymmetry induces symmetric divisions generating two outside cells, unlike in normal 8-cell stage embryos where the apical domain controls its asymmetric segregation (Korotkevich et al., 2017).

This mechanism would allow developing embryos to adjust cell division pattern flexibly as they undergo morphogenesis involving spatiotemporal variations, such as during early mammalian development (Dietrich and Hiiragi, 2007; Plusa et al., 2008; Saiz et al., 2016; Watanabe et al., 2014). More asymmetric divisions would generate fewer outside cells to stretch around the embryo surface while more cells accumulate inside, which in turn induce symmetric divisions driven by cell shape asymmetry. In contrast, fewer asymmetric divisions would generate more outside cells with a longer apico-basal axis, which in turn induce asymmetric divisions driven by the apical domain. These responses would ensure that developing embryos do not diverge too far away from an appropriate proportion of ICM and TE cells, possibly defined by geometrical constraints that is required for further development (Figure 7). In this way, the interplay between cell shape and polarity leads to system robustness in terms of tissue architecture and cell allocation, which in turn is important for cell fate specification.

This hypothesis was tested by experimental manipulation of the mouse embryo. When embryos are compressed and deformed, 16-cell embryos have fewer inner cells. However, upon release from deformation, the embryos rapidly increase the number of inner cells by asymmetric cell divisions and inward cell movement. We found that this recovery of inner cell number by asymmetric divisions is indeed accompanied by changes in cell geometry, in agreement with the hypothesis. Inward cell movement may well be induced by differential contractility inherited during the 8–16 cell division, a mechanism in operation at this stage of embryo (Maître et al., 2016; Samarage et al., 2015).

We have recently shown that fluid pressure in blastocyst cavities increases during blastocyst development, and that feedback between luminal pressure and tissue mechanics controls blastocyst size and cell fate allocation (Chan et al., 2019). Based on the compression experiment, we here propose another key role of the fluid-filled cavity—the pressurized cavity brings about the spherical shape of the blastocyst. Furthermore, cavity expansion in the late blastocyst would ensure that the outside cells are sufficiently stretched to divide symmetrically, such that no ectopic inside cells arise when TE and ICM cell fates are already determined (Posfai et al., 2017; Rossant and Lis, 1979; Rossant and Vijh, 1980). Taken all together, the tug-of-war mechanism of division orientation control, combined with control of embryonic size and shape by the lumen, ensures the robust establishment of the blastocyst architecture and pattern.

The control of division orientation by cell geometry and polarity is in line with a recent study in early ascidian embryos (Dumollard et al., 2017). In addition to cell shape change, the potential increase of cortical tension upon release from compression might also contribute to the control of spindle orientation (Campinho et al., 2013; Finegan et al., 2019; Scarpa et al., 2018; Wang et al., 2017). While the detailed molecular mechanisms that compete to control division orientation remain a topic

for future studies, it would be interesting to examine whether this tug-of-war type coordination exists in other tissues harboring regulative capacity.

STAR★METHODS

Detailed methods are provided in the online version of this paper and include the following:

- KEY RESOURCES TABLE
- LEAD CONTACT AND MATERIALS AVAILABILITY
- EXPERIMENTAL MODEL AND SUBJECT DETAILS
  - Animal Work
  - Mouse Lines and Genotyping
  - Recovery of Mouse Embryos
- METHOD DETAILS
  - *In Vitro* Transcription and Microinjection of mRNAs
  - Immunostaining
  - Confocal Microscopy
  - Spinning-Disk Microscopy
  - Light-Sheet Microscopy
  - Fabrication of The Compression Device
  - Isolation of Blastomeres from Embryos
  - Compression of Blastomeres and Embryos
- QUANTIFICATION AND STATISTICAL ANALYSIS
  - Image Analysis
  - Statistical Analysis
- DATA AND CODE AVAILABILITY

SUPPLEMENTAL INFORMATION

Supplemental Information can be found online at https://doi.org/10.1016/j.devcel.2019.10.012.

ACKNOWLEDGMENTS

We are grateful to members of the Hiiragi lab for discussions and comments on the manuscript and the EMBL animal facility for their support. We thank Ekaterina Korotkevich for Par-6-GFP mice, Ulla-Maj Fiuza for the comparison of image analysis methods, and Judith Reichmann and Petr Strnad for the help with SPIM imaging. R.N. is supported by fellowships from Marie Sklodowska-Curie Actions (grant number 326701) and from JSPS. D.F. is supported by the EMBL Interdisciplinary Postdoc Program (EIPOD) under Marie Curie Actions (COFUND III RTD). The Hiiragi laboratory is supported by EMBL, the German Research Foundation, and the European Research Council (ERC Advanced Grant "SelforganisingEmbryo," grant agreement 742732).

AUTHOR CONTRIBUTIONS

R.N., P.M., and T.H. designed the study; R.N. and P.M. conducted the experiments and interpreted the data together with T.H.; Y.-J.L. and M.P. developed the compression device; D.F. generated and analyzed some of the images; and F.B. generated BAC-transgenic ES cells expressing PARD6b-EGFP. R.N. and P.M. prepared figures, tables, videos, and methods, and T.H. wrote the manuscript with input from all authors.

DECLARATION OF INTERESTS

The authors declare no competing interests.

Received: March 10, 2019
Revised: July 4, 2019
Accepted: October 14, 2019
Published: November 14, 2019

REFERENCES

Abe, T., Kiyonari, H., Shioi, G., Inoue, K.I., Nakao, K., Aizawa, S., and Fujimori, T. (2011). Establishment of conditional reporter mouse lines at ROSA26 locus for live cell imaging. Genesis *49*, 579–590.

Aiken, C.E.M., Swoboda, P.P.L., Skepper, J.N., and Johnson, M.H. (2004). The direct measurement of embryogenic volume and nucleo-cytoplasmic ratio during mouse pre-implantation development. Reproduction *128*, 527–535.

Anani, S., Bhat, S., Honma-Yamanaka, N., Krawchuk, D., and Yamanaka, Y. (2014). Initiation of Hippo signaling is linked to polarity rather than to cell position in the pre-implantation mouse embryo. Development *141*, 2813–2824.

Bao, Z., Murray, J.I., Boyle, T., Ooi, S.L., Sandel, M.J., and Waterston, R.H. (2006). Automated cell lineage tracing in Caenorhabditis elegans. Proc. Natl. Acad. Sci. U S A *103*, 2707–2712.

Biggers, J.D., McGinnis, L.K., and Raffin, M. (2000). Amino acids and preimplantationdevelopment of the mouse in protein-free potassium simplex optimizedmedium. Biol. Reprod. *63*, 281–293.

Bosveld, F., Markova, O., Guirao, B., Martin, C., Wang, Z., Pierre, A., Balakireva, M., Gaugue, I., Ainslie, A., Christophorou, N., et al. (2016). Epithelial tricellular junctions act as interphase cell shape sensors to orient mitosis. Nature *530*, 495–498.

Boyle, T.J., Bao, Z., Murray, J.I., Araya, C.L., and Waterston, R.H. (2006). AceTree: a tool for visual analysis of Caenorhabditis elegans embryogenesis. In BMC Bioinformatics, *7*, K. Cockburn and S. Biechele, eds., p. 275.

Campinho, P., Behrndt, M., Ranft, J., Risler, T., Minc, N., and Heisenberg, C.P. (2013). Tension-oriented cell divisions limit anisotropic tissue tension in epithelial spreading during zebrafish epiboly. Nat. Cell Biol. *15*, 1405–1414.

Chan, C.J., Costanzo, M., Ruiz-Herrero, T., Monke, G., Petrie, R., Mahadevan, L., and Hiiragi, T. (2019). Hydraulic control of embryo size, tissue shape and cell fate. Nature *571*, 112–116.

Dietrich, J.E., and Hiiragi, T. (2007). Stochastic patterning in the mouse pre-implantation embryo. Development *134*, 4219–4231.

Dumollard, R., Minc, N., Salez, G., Aicha, S.B., Bekkouche, F., Hebras, C., Besnardeau, L., and McDougall, A. (2017). The invariant cleavage pattern displayed by ascidian embryos depends on spindle positioning along the cell's longest axis in the apical plane and relies on asynchronous cell divisions. ELife *6*, 495.

Fernandez, R., Das, P., Mirabet, V., Moscardi, E., Traas, J., Verdeil, J.L., Malandain, G., and Godin, C. (2010). Imaging plant growth in 4D: robust tissue reconstruction and lineaging at cell resolution. Nat. Methods *7*, 547–553.

Finegan, T.M., Na, D., Cammarota, C., Skeeters, A.V., Nádasi, T.J., Dawney, N.S., Fletcher, A.G., Oakes, P.W., and Bergstralh, D.T. (2019). Tissue tension and not interphase cell shape determines cell division orientation in the Drosophila follicular epithelium. EMBO J. *38*, e100072.

Graham, C.F., and Lehtonen, E. (1979). Formation and consequences of cell patterns in preimplantation mouse development. J. Embryol. Exp. Morphol. *49*, 277–294.

Hadjantonakis, A.K., and Papaioannou, V.E. (2004). Dynamic in vivo imaging and cell tracking using a histone fluorescent protein fusion in mice. BMC Biotechnol. *4*, 33.

Hebert, A.M., Duboff, B., Casaletto, J.B., Gladden, A.B., and McClatchey, A.I. (2012). Merlin/ERM proteins establish cortical asymmetry and centrosome position. Genes Dev. *26*, 2709–2723.

Hertwig, O., and Hertwig, R. (1884). Untersuchungen zur Morphologie und Physiologie der Zelle. Jenaische Z. Naturwiss. *18*, 276–318.

Johnson, M.H., and Ziomek, C.A. (1981). The foundation of two distinct cell lineages within the mouse morula. Cell *24*, 71–80.

Korotkevich, E., Niwayama, R., Courtois, A., Friese, S., Berger, N., Buchholz, F., and Hiiragi, T. (2017). The apical domain is required and sufficient for the first lineage segregation in the mouse embryo. Dev. Cell *40*, 235–247.

Kurotaki, Y., Hatta, K., Nakao, K., Nabeshima, Y.-I., and Fujimori, T. (2007). Blastocyst axis is specified independently of early cell lineage but aligns with the ZP shape. Science *316*, 719–723.

Le Berre, M., Aubertin, J., and Piel, M. (2012). Fine control of nuclear confinement identifies a threshold deformation leading to lamina rupture and induction of specific genes. Integr. Biol. (Camb) *4*, 1406–1414.

Maître, J.L., Niwayama, R., Turlier, H., Nédélec, F., and Hiiragi, T. (2015). Pulsatile cell-autonomous contractility drives compaction in the mouse embryo. Nat. Cell Biol. *17*, 849–855.

Maître, J.L., Turlier, H., Illukkumbura, R., Eismann, B., Niwayama, R., Nédélec, F., and Hiiragi, T. (2016). Asymmetric division of contractile domains couples cell positioning and fate specification. Nature *536*, 344–348.

Mikula, K., Peyriéras, N., Remešíková, M., and Stašová, O. (2011). Segmentation of 3D cell membrane images by PDE methods and its applications. Comput. Biol. Med. *41*, 326–339.

Minc, N., Burgess, D., and Chang, F. (2011). Influence of cell geometry on division-plane positioning. Cell *144*, 414–426.

Morris, S.A., Teo, R.T.Y., Li, H., Robson, P., Glover, D.M., and Zernicka-Goetz, M. (2010). Origin and formation of the first two distinct cell types of the inner cell mass in the mouse embryo. Proc. Natl. Acad. Sci. USA *107*, 6364–6369.

Muzumdar, M.D., Tasic, B., Miyamichi, K., Li, L., and Luo, L. (2007). A global double-fluorescent Cre reporter mouse. Genesis *45*, 593–605.

Pierre, A., Sallé, J., Wühr, M., and Minc, N. (2016). Generic theoretical models to predict division patterns of cleaving embryos. Dev. Cell *39*, 667–682.

Plusa, B., Piliszek, A., Frankenberg, S., Artus, J., and Hadjantonakis, A.K. (2008). Distinct sequential cell behaviours direct primitive endoderm formation in the mouse blastocyst. Development *135*, 3081–3091.

Poser, I., Sarov, M., Hutchins, J.R.A., Hériché, J.K., Toyoda, Y., Pozniakovsky, A., Weigl, D., Nitzsche, A., Hegemann, B., Bird, A.W., et al. (2008). BAC TransgeneOmics: a high-throughput method for exploration of protein function in mammals. Nat. Methods *5*, 409–415.

Posfai, E., Petropoulos, S., de Barros, F.R.O., Schell, J.P., Jurisica, I., Sandberg, R., Lanner, F., and Rossant, J. (2017). Position- and Hippo signaling-dependent plasticity during lineage segregation in the early mouse embryo. ELife *6*, e22906.

Rabut, G., and Ellenberg, J. (2004). Automatic real-time three-dimensional cell tracking by fluorescence microscopy. J. Microsc. *216*, 131–137.

Rossant, J., and Lis, W.T. (1979). Potential of isolated mouse inner cell masses to form trophectoderm derivatives in vivo. Dev. Biol. *70*, 255–261.

Rossant, J., and Tam, P.P.L. (2009). Blastocyst lineage formation, early embryonic asymmetries and axis patterning in the mouse. Development *136*, 701–713.

Rossant, J., and Vijh, K.M. (1980). Ability of outside cells from preimplantation mouse embryos to form inner cell mass derivatives. Dev. Biol. *76*, 475–482.

Saiz, N., Williams, K.M., Seshan, V.E., and Hadjantonakis, A.K. (2016). Asynchronous fate decisions by single cells collectively ensure consistent lineage composition in the mouse blastocyst. Nat. Commun. *7*, 13463.

Samarage, C.R., White, M.D., Álvarez, Y.D., Fierro-González, J.C., Henon, Y., Jesudason, E.C., Bissiere, S., Fouras, A., and Plachta, N. (2015). Cortical tension allocates the first inner cells of the mammalian embryo. Dev. Cell *34*, 435–447.

Santella, A., Du, Z., Nowotschin, S., Hadjantonakis, A.K., and Bao, Z. (2010). A hybrid blob-slice model for accurate and efficient detection of fluorescence labeled nuclei in 3D. BMC Bioinformatics *11*, 580.

Scarpa, E., Finet, C., Blanchard, G.B., and Sanson, B. (2018). Actomyosin-driven tension at compartmental boundaries orients cell division independently of cell geometry in vivo. Dev. Cell *47*, 727–740.

Schindelin, J., Arganda-Carreras, I., Frise, E., Kaynig, V., Longair, M., Pietzsch, T., Preibisch, S., Rueden, C., Saalfeld, S., Schmid, B., Tinevez, J.Y., White, D.J., Hartenstein, V., Eliceiri, K., Tomancak, P., and Cardona, A. (2012). Fiji: an open-source platform for biological-image analysis. Nat. Methods *9*, 676–682.

Sommer, C., Straehle, C., Kothe, U., and Hamprecht, F.A. (2011). In: Ilastik: Interactive Learning and Segmentation Toolkit (IEEE), pp. 230–233.

Strnad, P., Gunther, S., Reichmann, J., Krzic, U., Balazs, B., de Medeiros, G., Norlin, N., Hiiragi, T., Hufnagel, L., and Ellenberg, J. (2016). Inverted light-sheet microscope for imaging mouse pre-implantation development. Nat. Methods *13*, 139–142.

Tarkowski, A.K., and Wróblewska, J. (1967). Development of blastomeres of mouse eggs isolated at the 4- and 8-cell stage. J. Embryol. Exp. Morphol. *18*, 155–180.

Testa, G., Zhang, Y., Vintersten, K., Benes, V., Pijnappel, W.W.M.P., Chambers, I., Smith, A.J.H., Smith, A.G., and Stewart, A.F. (2003). Engineering the mouse genome with bacterial artificial chromosomes to createmultipurpose alleles. Nat. Biotechnol. *21*, 443–447.

Wang, M.F.Z., Hunter, M.V., Wang, G., McFaul, C., Yip, C.M., and Fernandez-Gonzalez, R. (2017). Automated cell tracking identifies mechanically oriented cell divisions during Drosophila axis elongation. Development *144*, 1350–1361.

Watanabe, T., Biggins, J.S., Tannan, N.B., and Srinivas, S. (2014). Limited predictive value of blastomere angle of division in trophectoderm and inner cell mass specification. Development *141*, 2279–2288.

Yamanaka, Y., Ralston, A., Stephenson, R.O., and Rossant, J. (2006). Cell and molecular regulation of the mouse blastocyst. Dev. Dyn. *235*, 2301–2314.

STAR★METHODS

KEY RESOURCES TABLE

Reagent or Resource	Source	Identifier
Antibodies		
Mouse Anti-Cdx2	BioGenex	MU392A-UC; RRID: AB_2335627
Anti-Sox2	R&D Systems,	AF2018; RRID: AB_355110
Chemicals, Peptides, and Recombinant Proteins		
Pregnant Mare's Serum Gonadotropin	Intervet	Intergonan
Human Chorionic Gonadotropin	Intervet	Ovogest 1500
KSOMaa	Zenith biotech	ZEKS-050
KSOMaa with HEPES	Zenith biotech	ZEHP-050
KSOMaa without Ca2+ and Mg2+ Custom-Made N/A	Custom-made	N/A
Mineral Oil	Sigma	M8410
Hyaluronidase	Sigma	H4272
Pluronic® F-127	Sigma	P2443-250G
Fibronectin	Sigma	F4759
Trimethylchlorosilane	Sigma	386529–25ML
Sylgard 184 1,1 kg Kit	Dow Corning	N/A
SU-8 2025	MicroChem	Y111069
SU-8 2015	MicroChem	Y111064
PVP-40	Sigma	P0930
Critical Commercial Assays		
mMessage m Machine Transcription Kit	Ambion	AM1348
Experimental Models: Cells		
R1/E ES Cells	Transgenic Core Facility at Max Planck Insititute of Molecular ell Biology and Genetics	N/A
Experimental Models: Organisms/Strains		
Mouse: (C57BL/6xC3H) F1	Laboratory Animal Resources at the European Molecular Biology Laboratory	N/A
Mouse: R26-H2B-MCherry	Laboratories of Animal Resource development and Genetic Engineering, RIKEN Center for Life Science Technologies; Abe et al., 2011	CDB0239K
Mouse: mTmG	The Jackson Laboratory; Muzumdar et al., 2007	007676
Mouse: mG	The Jackson Laboratory; Muzumdar et al., 2007	007676
H2B-EGFP	Hadjantonakis and Papaioannou, 2004	006069
Mouse: Pard6b-GFP BAC Transgenic	This study	N/A
Recombinant DNA		
pRN3-Ezrin-GFP	S. Louvet-Vallée	N/A
BAC#HS.E052.F15 (for Pard6b-GFP)	BACPAC Resource Center (BPRC)	N/A
LAP Tagging Cassette	Poser et al., 2008	N/A
Oligonucleotides		
See Table S1 for Genotyping Primer List	N/A	N/A
Software and Algorithms		
MATLAB	MathWorks	https://www.mathworks.com/products/matlab.html
R	N/A	https://www.r-project.org
Fiji	Schindelin et al., 2012	https://fiji.sc

(*Continued on next page*)

Continued		
Reagent or Resource	Source	Identifier
Imaris	BITPLANE	https://imaris.oxinst.com
Ilastik	Sommer et al., 2011	http://ilastik.org
StarryNite	Bao et al., 2006; Santella et al., 2010	http://starrynite.sourceforge.net
AceTree	Boyle et al., 2006	http://starrynite.sourceforge.net
Python	N/A	https://www.python.org/
AutofocusScreen	Rabut and Ellenberg, 2004	https://www-ellenberg.embl.de/resources/microscopyautomation
C++	N/A	N/A
Point Cloud Library	PointCloudLibrary	http://www.pointclouds.org
Sparse Field Methods for Active Contours	Shawn Lankton	https://www.mathworks.com/matlabcentral/fileexchange/23847-sparse-field-methods-for-active-contours

LEAD CONTACT AND MATERIALS AVAILABILITY

Further information and requests for resources and reagents should be directed to and will be fulfilled by the Lead Contact, Takashi Hiiragi (hiiragi@embl.de). Mouse lines generated in this study are available from the Lead Contact with a completed Materials Transfer Agreement.

EXPERIMENTAL MODEL AND SUBJECT DETAILS

Animal Work

We performed all animal work in the Laboratory Animal Resources at the European Molecular Biology Laboratory under the permission from institutional veterinarians overseeing operations (ARC number TH11 00 11). LAR is operated as stated in international animal welfare rules (Federation for Laboratory Animal Science Associations guidelines and recommendations). Mouse colonies are maintained in specific pathogen-free conditions with 12–12 h light-dark cycle. All mice used for experiments were older than 6 weeks.

Mouse Lines and Genotyping

The following mouse lines were used in this study: (C57BL/6xC3H) F1 for WT, mTmG (Muzumdar et al., 2007), R26-H2B-mCherry (Abe et al., 2011), H2B-EGFP (Hadjantonakis and Papaioannou, 2004) and PARD6b-EGFP BAC generated in this study.

PARD6b-EGFP BAC mice were mated with mTmG mice to quantify PARD6b-EGFP BAC signal intensity after 16-to-32 cell divisions (Figures 1A and 4A). To track cell shape and cell division orientation, mG mice were mated with R26-H2B-mCherry mice (Figures 2 and 3). WT or H2B-GFP mice were mated with mTmG transgenic mice to visualize plasma membranes (Figures 4B and 4C) or plasma membrane and nuclei (Figure 6), respectively. Sequences of oligos for genotyping PCR is written in Table S1.

To generate PARD6b-EGFP mice, the PARD6b gene was modified on a bacterial artificial chromosome by recombineering (Testa et al., 2003). The stop codon of the PARD6b coding sequence in the RP11-723F14-BAC was replaced with the LAP cassette (Poser et al., 2008). The LAP tagging cassette consists of EGFP sequence followed by an internal ribosome entry site and the neomycin-kanamycin resistance gene for eukaryotic and bacterial expression. A correct placement of the tagging cassette was confirmed by PCR amplifying the integration site using 5′-AGCTTTGAGCCAGAGGATGA (hPARD6b- and 5′-GCCTATTCCACGTCACTGGT (hPARD6b-R) primers producing a 3,500 bp fragment. To generate a transgenic ES cell line, the modified BAC was transfected into R1/E ES cells that were selected for BAC integration with 250 mg/ml G418 (Invitrogen, 10131-019). The ES cells were subsequently injected into C57BL/6 blastocysts that were transferred into pseudo-pregnant CD1 female mice. The resultant pups were examined for the presence of BAC integration by genotyping.

Recovery of Mouse Embryos

Embryos were recovered from superovulated female mice. For superovulation, intraperitoneal injection of 5 international units (IU) of pregnant mare's serum gonadotropin (PMSG, Intervet Intergonan) and following injection of 5-IU human chorionic gonadotropin (hCG; Intervet, Ovogest 1500) 48 h later were performed. Zygotes were recovered at E = 0.5 by opening ampulla in KSOMaa, including HEPES (H-KSOMaa; Zenith Biotech, ZEHP-050) supplemented with 300 μg/ml hyaluronidase (Sigma, H4272) and 10 mg/ml PVP-40 (Sigma, P0930). Two-cell stage embryos were recovered by flushing oviducts with H-KSOMaa at E1.5. Recovered embryos were washed three times in H-KSOMaa drops and then transferred into 10 μl drops of KSOMaa (Zenith Biotech, ZEKS-050) covered with mineral oil (Sigma, M8410). Embryos were cultured in an incubator (Thermo Scientific) at 37°C with, the supply of 5% CO_2.

METHOD DETAILS

In Vitro Transcription and Microinjection of mRNAs

In vitro transcription of Ezrin-GFP mRNA from pRN3-Ezrin-GFP plasmid was performed using mMessage mMachine transcription kit (AM1348) as described in Korotkevich et al. (2017).

Microinjection was performed with an injector (Epperndorf, FemtoJet) and micromanipulators (Narishige, MON202-D) mounted on inverted epifluorescence microscope (Zeiss, Axio Observer.Z1). The incubation chamber on the microscope was kept at 33.5°C during microinjection. Injection needles were made by pulling capillaries (Warner Instruments, GC100TF-15) using a needle puller (Sutter Instrument, P-97) and bending their tips with a microforge (Narishige, MF-900). mRNAs were injected to the cytoplasm of zygotes at 22 h post-hCG, which were kept in a drop of 10 μl of H-KSOMaa covered with mineral oil. Before injection, RNA solution was centrifuged with 5,000g for 15 min at 4°C.

Immunostaining

Embryos were washed in 3 drops of 50 μl of DPBS. Then, they were fixed in 4% paraformaldehyde (PFA, Electron Microscopy Sciences, 19208) in DPBS for 15 min at 37°C, washed in DPBS with 0.1% Tween-20 (DPBSw, Sigma, P7949), permeabilized in 0.5% TritonX-100 (Sigma, T8787) in DPBS for 35 min and blocked at 4°C overnight in DPBSw with 5% bovine serum albumin (BSA, Sigma, 9647). Embryos were then transferred into 50 μl of primary antibody solution in DPBSw with 3% BSA and incubated overnight at 4°C. Primary antibodies against Cdx2 (Biogenex, MU392A-UC) and Sox2 (R&D Systems, AF2018) were diluted at 1:100. Secondary antibodies conjugated with Cy5 and targeted to mouse Ig (Jackson ImmunoResearch), 715-715-150) and ones conjugated with Alexa Fluor 488 targeted to Goat Ig(Lifetechnologies, A11005) were diluted at 1:200. Before imaging, embryos were transferred and incubated for 2 h in 50 μl of secondary antibody solution at room temperature. Embryos were then washed 3 times in DPBS, and mounted in DPBS with DAPI (1:2,000, Invitrogen, D3751) to stain DNA.

Confocal Microscopy

Imaging of immunostained embryos (Figure 5) and live-imaging of embryos expressing fluorescent markers (Figures 1 and 6) was performed with LSM 780 (Zeiss). C-Apochromat 40× 1.1 NA water objective (Zeiss) was used. For live imaging, temperature and CO_2 concentration were maintained at 37°C and 5% respectively. Tracking of embryos during live-imaging to compensate for sample drift on the stage was done using an automatic real-time 3D cell tracking macro, AutofocusScreen (available at http://www.ellenberg.embl.de/index.php/software/microscopyautomation) (Rabut and Ellenberg, 2004).

Spinning-Disk Microscopy

Inverted Zeiss Observer Z1 installed with a CSU-X1M 5000 spinning-disk unit was used for compression of whole embryos and dissociated cells. 488 nm and 561 nm laser beams were used for the excitation. 63× 1.2 NA water immersion objective was used. Emission light went through 525/50 nm or 629/62 nm band pass filter and was imaged on EMCCD camera (Photometrics, Evolve 512). The microscope was operated with Zen software. Temperature and CO_2 concentration were maintained 37°C and 5% respectively. Images were taken every 10 min.

Light-Sheet Microscopy

Inverted light-sheet microscope developed at EMBL (Strnad et al., 2016) was used for live-imaging mouse embryos (Figures 2 and 3) and control embryos in Figure 6. To illuminate cell membrane more uniformly, a rotating 6 mm thick and 25-mm diameter glass plate (Thorlabs) was inserted in the illumination path. 488 nm and 561 nm laser beams passing through this mirror was translated by it at the back focal plane of the illumination objective 10 x 0.3 NA (Nikon, CFI Plan Fluor 10XW). Emission light was collected into 100 × 1.1 NA water immersion objective (Nikon, CFI Plan 100XW) and imaged on a CMOS camera (Hamamatsu Photonics, Orca Flash 4 V2). Images were taken every 10–15 min. The mounting of embryos on the microscope was done as described in Strnad et al. (2016). Temperature and CO_2 concentration were maintained 37°C and 5% respectively. The axial distance between two consecutive Z planes was set to 1 μm.

Fabrication of The Compression Device

The compression device used in this study was developed earlier (Le Berre et al., 2012). It is composed of a suction cup and a structured confining glass slide, which can be used on a standard petri dish compatible with fluorescence microscopy. In brief, the confining structure with micropillars on the coverslips was made of PDMS, casted from molds fabricated by standard photolithography. Either photoresist SU8 2015 or SU8 2025 (MicroChem) was chosen to fabricate the mold on a silicon wafer with a regular microholes array (diameter: 440 μm, 1 mm spacing), depending on the heights of micropillars, which is either 16 μm or 29 μm in this paper.

Before pouring PDMS, the manufactured mold was treated with trimethylcholorosilane (TMCS, Sigma, 386529–25ML) for 5 min by evaporation. Afterwards, a mixture of PDMS (8/1 w/w PDMS A/cross linker B) was degassed by centrifugation at 4,000 rpm for 3 min, and poured into the SU8 mold. Then, a 10-mm standard microscopy coverslip, freshly activated for 2 min inside a plasma cleaner (PlasmaPrep 2), was pressed on PDMS (the activated side facing to PDMS) to get a minimal thickness of PDMS layer. After baking at 95°C on a hot plate for 15 min, excessive PDMS was removed from the mold. In order to facilitate the detachment of coverslips with

PDMS micro-structures from the mold, a drop of isopropanol was added on the slide. Finally, the slide was gently raised by inserting a razor blade between the slide and the mold, allowing the confining coverslips with the structured PDMS micropillars to be lifted away.

Isolation of Blastomeres from Embryos

To isolate single blastomeres, embryos were placed into a pronase drop covered with mineral oil (0.5% w/v Proteinase K in H-KSOMaa supplemented with 0.5% PVP-40) for 2 min. Embryos were then washed in 7 drops of 10 μl KSOMaa-HEPES. Afterwards, the embryos were placed into a 50 μl of drop of KSOMaa without Ca2+ and Mg2+ (Biggers et al., 2000). Blastomeres were then dissociated in the drop by pipetting up and down in a glass capillary (Brand, 708744). Dissociated blastomeres were incubated in KSOMaa-HEPES drops covered with mineral oil.

Compression of Blastomeres and Embryos

The confining suction cup was rinsed for one minute in 15 ml 100% ethanol, 3 min in 40 ml DPBS and then for overnight in KSOMaa with HEPES. It was kept until an hour before the compression and then transferred into the chamber of microscope where CO_2 concentration and temperature were maintained at 5% and 37°C, respectively. A coverslip with micropillars were treated for one minute in plasma cleaner and put into a petri dish while keeping the side with micropillars up. Then, 50 μl of 100-μg/ml Pluronic (Sigma, P2443-250) dissolved in PBS was placed on the confining slide. The petri dish was covered and wrapped with a sheet of parafilm to prevent the evaporation of Pluronic, and kept at 4°C until 20 min before the compression experiment. The imaging dish (Mattek, P60G-1.5-30-F) was activated inside plasma cleaner for a minute. Then 1.0 to 1.2 ml of KSOMaa with HEPES followed by 2 ml of mineral oil was placed on the dish. The fluid connector tip and vacuum tube was connected to a microfluidic pressure controller (Fluigent, MFCS-VAC) and a microfluidic pump. Custom-made Labview (National Instrument) software, Dikeria, was used to control the pressure. Embryos or cells were placed at the center of imaging dish. Then, the dish was placed on the microscope stage. After that, the fluid connecter was inserted into the suction cup, and the confining coverslip with micropillars was attached on the piston of suction cup. By using Dikeria, initially the pressure was controlled to be ~7 mbar. We put the suction cup on the dish and increased the pressure to ~35–50 mbar to compress embryos till the specified height (30μm for compression in Figure 4C and 65μm for that in Figure S3), and ~60 mbar to compress isolated cells.

QUANTIFICATION AND STATISTICAL ANALYSIS

Image Analysis

Lineage Tracking

Images of nuclei channel and membrane channel were cropped from raw images of the light-sheet microscope where both channels were imaged on two distinct positions of a image. Both channels were resized so that a voxels size become 0.5 × 0.5 × 0.5 μm. Subsequently, images of nuclei channel were converted into 8-bit with Fiji while membrane images were smoothed by GradientAnisotropicDiffusionImageFilter of ITK library and then converted into 8-bit using Fiji. To segment nuclei, a composite 8-bit image of both channels were created and converted into RGB image by Fiji. The RGB image was trained with machine learning algorithm of Ilastik to segment the voxels with nuclei signals. Coordinates of the centroids of individual nuclei were then obtained from the probability map output of Ilastik using 3D Objects Counter of Fiji. To facilitate the tracking of nuclei, drifts and rotations of embryos in the image were compensated by aligning coordinates of nuclei using point cloud library (pcl). Using the transformation matrix output from pcl and Fiji, aligned images, where no drifts or no rotations of embryos occur, were obtained. By applying StarryNite (Bao et al., 2006; Santella et al., 2010) and AceTree (Boyle et al., 2006), the nuclei in aligned images were tracked. After the tracking of nuclei in aligned images, the positions of nuclei in original images were recovered with the inverses of transformation matrixes.

Segmentation of the Embryonic Surface

After the conversion into 8-bit images of membrane channel (see *Lineage tracking*), these images were binarized to obtain an approximative outer surface of embryos using Fiji. The threshold value of the binarization was chosen so that the approximative surface contains the entire embryo and therefore bigger than the embryo. Then, more precise surface was obtained by applying Level set. As an initial condition of Level set, the voxels where $\phi = 0$ were set the approximative surface.

Our algorithm of Level set was based on the generalized subjective surface (GSUBSURF) equation (14) in Mikula et al. (2011):

$$\phi_t - w_a \nabla g . \nabla \phi - w_d g |\nabla \phi| \nabla . \left(\frac{\nabla \phi}{|\nabla \phi|} \right) = 0$$

but following points were new; 1) To accelerate the algorithm, sparse field method was adopted (R. Whitaker. A level-set approach to 3D reconstruction from range data. International Journal of Computer Vision, 29(3):203-231, 1998.). 2) The ballooning term, $\delta g|\nabla\phi|$, was added to the right side of the equation (14), where δ is a constant number, g is an edge detector function as written in Mikula et al. (2011), and $|\nabla\phi|$ is the length of the gradient vector of the Level set function ϕ. 3) The equation was solved with SOR method.

Starting from the approximative surface, the voxels where $\phi = 0$ evolved inwards and stopped at the cell-medium interface, giving a more precisely segmented embryonic surface. As this boundary localizes 1~2 voxel outside the membrane signal. The final counter, which overlaps with the positions of cell membrane signal, was obtained by shrinking the boundary using Fiji's Erode (3D) function.

Membrane Segmentation and Measurement of Geometric Parameters

The voxels with membrane signal were segmented by Ilastik from 8-bit images, which generated the probability map. Our Level set was applied to this probability map. As an initial condition, the voxels where $\phi = 0$ were set a small region surrounding the centroid of the nucleus of individual cell, which was detected in lineage tracing (Figure 2A). For images without nuclei signal (Figures 1A and 4A), the region corresponding to $\phi = 0$ at the beginning of the calculation was selected manually by specifying a point near the center of the cytoplasm. By solving the equation of Level set, the voxels where $\phi = 0$ enlarged in the cytoplasm and stopped when they reached the cell membrane, giving the border between the cytoplasm and the membrane. The zero points of ϕ converged into cell membrane in 99.6% of segmentations of images data for Figure 3. In Video S2, for cells in which the zero points did not converge into a solution, we depicted 5×5 pixels square at the location of their nuclei. The border localized at the edge of the cytoplasm was further enlarged by Dilate (3D) functions until it overlapped the cell membrane. This caused overlaps among adjacent cells, but these were resolved by calculating the 3D distances from voxels $\phi = 0$ and allocating the overlapping voxels to a cell that had the minimum distance. The outer surface of embryos was kept as obtained in *Segmentation of the embryonic surface*.

From thus obtained cellular segmentations, cell volume, V, was obtained by 3D Objects Counter of Fiji. To calculate the aspect ratio of cells, the matrix of the momentum of inertia was calculated at first, from which principal moments of inertia and their eigenvectors were obtained. The orientations of long, medium, short axes were given by the orientation of eigenvectors for smallest, medium, highest values of principal moments of inertia, (I_1, I_2, I_3), respectively. The radii of long, medium and short axes, (r_1,r_2,r_3) were given by sqrt(5/2×(I_2+I_3-I_1)/V), sqrt(5/2×(I_3+I_1-I_2)/V), sqrt(5/2×(I_1+I_2-I_3)/V), where sqrt means the square root. The aspect ratio was given by r_1/r_3.

Segmentation of Embryonic Cavities

The probability map of the cell membrane acquired by Ilastik was resized so that the voxel size becomes 1 × 1 × 1 μm. Gaussian blur 3D was applied to images, which were then inverted by Fiji. Using 3D Object Counter in Fiji, the cavity size was measured.

Division Angle

A unit vector along the orientation of the shortest axis at 50 min before division, **e1**, was detected as described in *Membrane segmentation and measurements of geometric parameters*. Another unit vector, **e2**, along the axis connecting centroids of two daughter cells just after division was also acquired to calculate inner product e1•e2, whose absolute value was transformed by arccosine function to obtain the angle. Therefore, acquired angles between 0 and 90 degrees were subtracted from 90 degrees, which gave the θ in Figure 3C. The random distribution of thus acquired angles follows sine functions, and the data in Figure 3C were graphically normalized so that the random distribution shows equal lengths of triangles in each data range.

To analyze the relationship between cell shape and the apico-basal axis in the 8-cell embryo (Figure S2), the longest axis of a cell was defined 60 min before division, whereas its apico-basal axis was approximated as the axis connecting the center of mass of the embryo and that of individual cells at the same time. The angle between the two axes was calculated from the inner product of the unit vectors along the two axes.

Quantification of PARD6b-EGFP Signals

Images were resized so that the voxel size becomes 0.5 × 0.5 × 2.5 μm. Images of membrane channel were converted to 8-bit image. Voxels belonging to cell membrane were trained with Ilastik, which generated the probability map. Our Level set was applied to the probability map, which gives the border between the cytoplasm and the cell membrane, **b1**. **b1** was enlarged to obtain the border containing outer surface of the cell membrane, **b2**, which was done by using Dilate function of Fiji. The region bigger than b1 and smaller than b2, thus, gave the region where membrane signal exists. The Pard6b GFP signal in this region was summed up over the voxels that belong to this region. Using Gaussian mixture model of python, the region where the background signal exists and one where strong Pard6b-GFP signal exists were separated in each cell. The total signal intensities from latter regions of two sister cells were summed up and their ratio was calculated. This quantification was done 10 min after the division of the mother cell.

Quantification of Ezrin-GFP Signals

Images were resized so that the voxel size becomes 0.5 × 0.5 × 1 μm. The apical domain marked with Ezrin-GFP was directly segmented by Ilastik, and then the total intensity of the Ezrin-GFP signal of two daughter cells was quantified, from which the signal intensity ratio was calculated. Considering the characteristics of Ezrin-GFP that its signal accumulates at the cleavage furrow during cytokinesis, the quantification was done after an hour the cytokinesis (Korotkevich et al., 2017).

Quantification of Sox2 and Cdx2 Signals in Immunostained Embryos

The intensity of both Sox2 and Cdx2 signals were quantified using Fiji. In detail, single Z plane was selected for individual nucleus, and the nucleus was segmented manually with polygon tool of Fiji. The Sox2 and Cdx2 signal intensity in the segmented region was quantified. The ratio of Sox2 signal to Cdx2 signal was then calculated for individual nucleus, and the statistics, from which dividing cells were excluded, of the ratio was fitted with Gaussian mixture model using Python. This classifies nuclei into those with higher Sox2/Cdx2-signal intensity ratio and those with lower ratio. Cells with nuclei that belong to the group with higher ratio were classified as Sox2 positive cells whereas the other nuclei were classified Cdx2 positive cells.

Statistical Analysis

Statistical analysis was performed using Python 3.0 (https://www.python.org/) for Student's t-test and Kolmogorov-Smirnov test. R (https://www.r-project.org) was used for the Dunnett's test to compare the aspect ratio between 16-cell outside cells and the other data (Figure 3A). No statistical analysis was used to predetermine sample size. Sample sizes, statistical tests and p-values are indicated in the text, figures and figure legends. n-values indicate number of embryos analyzed for different experimental conditions unless mentioned otherwise, and error bars indicate mean ± SD.

DATA AND CODE AVAILABILITY

The datasets generated from live-imaging of developing embryos using InVi-SPIM are available upon request. Codes for embryo surface and cell segmentation (version 0.0.0) generated during this study are available at the online repository: https://github.com/RitsuyaNiwayama

Developmental Cell, Volume *51*

Supplemental Information

A Tug-of-War between Cell Shape and Polarity Controls Division Orientation to Ensure Robust Patterning in the Mouse Blastocyst

Ritsuya Niwayama, Prachiti Moghe, Yan-Jun Liu, Dimitri Fabrèges, Frank Buchholz, Matthieu Piel, and Takashi Hiiragi

Supplemental Figures

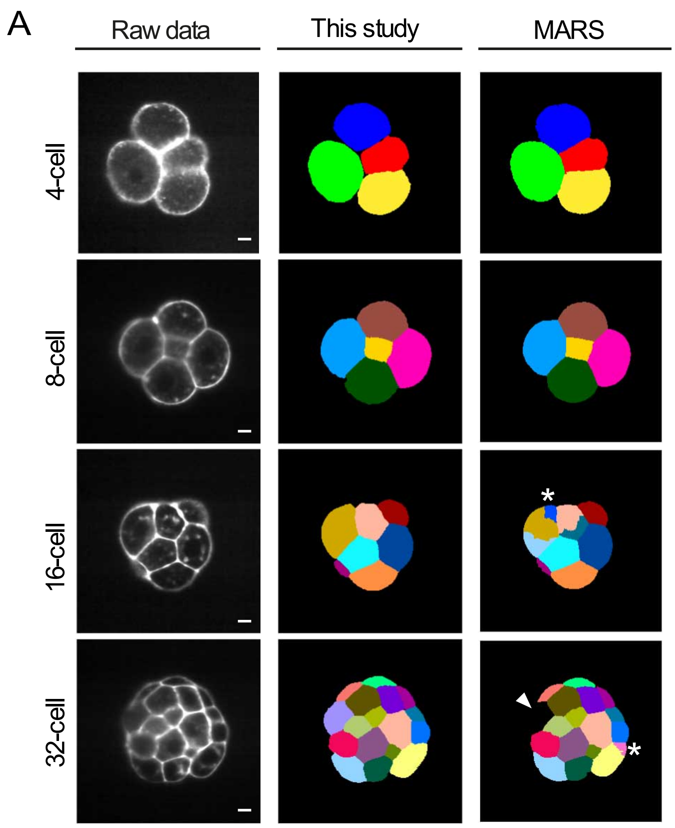

Figure S1. The fidelity of the cell membrane segmentation in this study, related to Figure 2

Representative images of the automatic membrane segmentation by the pipeline in this study and by MARS. Overall, the fidelity is comparable, with cases in which this pipeline is more optimal for segmenting the cell membrane of mouse embryos without having under-segmentation (arrowhead) or over-segmentation (stars). Scale bars: 10 μm.

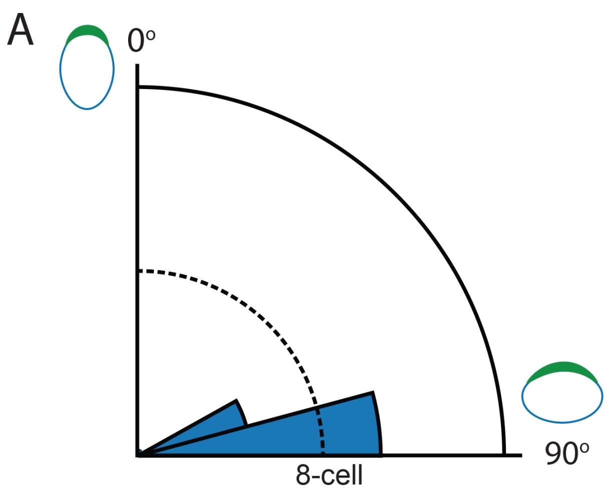

Figure S2. Relationship between cell shape and the apical domain in the 8-cell blastomeres, related to Figure 3

Distribution of the angle between the longest axis and apico-basal axis of the blastomeres in the 8-cell embryo. The solid curve represents the frequency of 1 and the dotted curve marks 0.5, with the frequency normalised to the total sample size (n=56 cells from 7 embryos), similarly to Figure 3C. While cell shape change is minimum in the 8-cell blastomeres (see Figure 3C), the apico-basal axis and the longest axis of the blastomeres are not aligned, rather they are typically perpendicular to each other. Therefore, we can clearly distinguish the impact of cell shape and the apical domain on the 8-to-16 cell divisions and conclude that it is the apical domain, but not cell shape, that controls spindle orientation during these divisions.

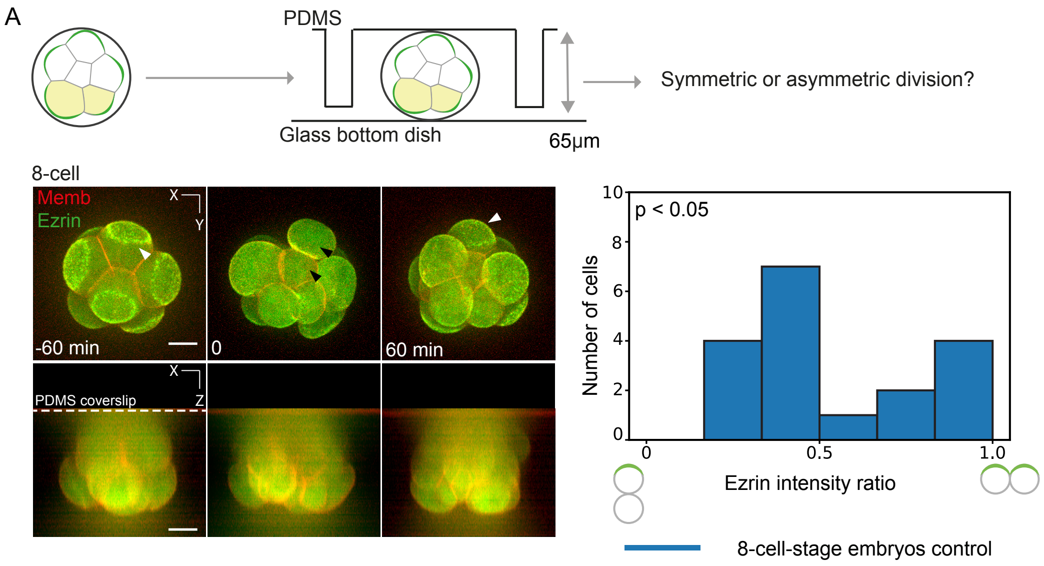

Figure S3. Cell compression of the 8-cell embryo does not change division orientation, related to Figure 4

Time-lapse images of a developing 8-cell embryo expressing Ezrin-GFP (green) and mT (red). The distribution of the Ezrin intensity ratio is significantly different from that of compressed cells in Figure 4C. p=0.039, n = 18 cells from N = 6 embryos, Kolmogorov-Smirnov test. The height of compressed embryos is 65 μm. Time 0 at the cytokinesis. White arrowheads, the apical domain. Black arrowheads, daughter cells after division. Dashed line, the compression plates. Scale bar, 10 μm.

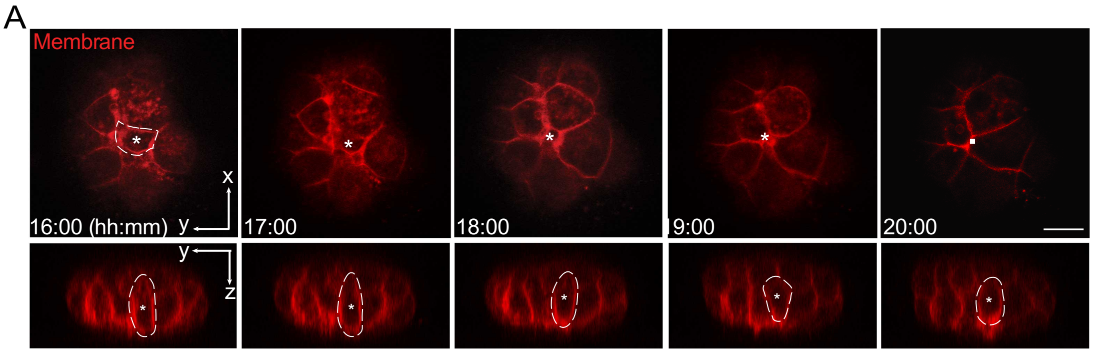

Figure S4. Cell movement as a mechanism of cell fate allocation in the blastocyst during recovery of spherical shape of the embryo, related to Figure 6

Representative images of embryos expressing mT (red) showing inward movement of cells during recovery of spherical shape of the embryo after releasing compression, contributing to inside cells. Scale bar: 20 µm.

Mouse line	Primer name	Source	Sequence	PCR product size (bp)
mTmG and mG	oIMR7318	The Jackson Laboratory, Muzumdar et al., 2007	CTCTGCTGCCTCCTGGCTTCT	Knock-in allele = 250 WT allele = 330
	oIMR7319		CGAGGCGGATCACAAGCAATA	
	oIMR7320		TCAATGGGCGGGGGTCGTT	
R26-H2B-mCherry	R26-P3	Abe et al., 2011	TCCCTCGTGATCTGCAACTCCAGTC	WT allele = 217 Knock-in allele = 270
	R26-P4		AACCCCAGATGACTACCTATCCTCC	
	R26-P6		GCTGCAGGTCGAGGGACC	
Pard6b GFP BAC	Pard6b BAC fw4	This study	TTCATATCTCGGCTCGTCCC	686
	BAC rev		AAGTCGTGCTGCTTCATGTG	
H2B-GFP	GFP-qPCR2-F	This study	CACATGAAGCAGCACGACTT	440
	GFP-qPCR3-R		CCAAGCTGAAGGTGACCAAG	

Supplemental Tables

Table S1. Genotyping primers, related to STAR Methods
